# Testing Local Adaptation in a Natural Great Tit-Malaria System: An Experimental Approach

**DOI:** 10.1371/journal.pone.0141391

**Published:** 2015-11-10

**Authors:** Tania Jenkins, Jessica Delhaye, Philippe Christe

**Affiliations:** Department of Ecology and Evolution, University of Lausanne, Lausanne, Switzerland; Institute of Tropical Medicine, JAPAN

## Abstract

Finding out whether *Plasmodium* spp. are coevolving with their vertebrate hosts is of both theoretical and applied interest and can influence our understanding of the effects and dynamics of malaria infection. In this study, we tested for local adaptation as a signature of coevolution between malaria blood parasites, *Plasmodium* spp. and its host, the great tit, *Parus major*. We conducted a reciprocal transplant experiment of birds in the field, where we exposed birds from two populations to *Plasmodium* parasites. This experimental set-up also provided a unique opportunity to study the natural history of malaria infection in the wild and to assess the effects of primary malaria infection on juvenile birds. We present three main findings: i) there was no support for local adaptation; ii) there was a male-biased infection rate; iii) infection occurred towards the end of the summer and differed between sites. There were also site-specific effects of malaria infection on the hosts. Taken together, we present one of the few experimental studies of parasite-host local adaptation in a natural malaria system, and our results shed light on the effects of avian malaria infection in the wild.

## Introduction

Coevolution has inspired naturalists and evolutionary biologists since Darwin [1] and can be defined as the process of reciprocal selection between two interacting species leading to adaptation and counter-adaptation [2]. In host-parasite systems, the nature of coevolutionary interactions: e.g arms-race versus negative frequency dependent dynamics [3] can influence the evolution of virulence, and so affect the severity of both human and veterinary diseases [4]. Malaria, caused by *Plasmodium* spp., threatens about half the world’s population [5] and is one of the main sources of recent selection in human populations [6] with several well-studied genetic signatures of adaptation to malaria (e.g. mutations resulting in sickle cell anemia and the Duffy negative blood group reviewed in [7]). Though coevolution has been inferred from such genetic signatures, there is still very little experimental data on the nature and scale of coevolution among malaria parasites and their hosts, partly due to the difficult nature of designing such experiments involving malaria parasites.

A signature of coevolution in host-parasite systems is local adaptation (LA) of a parasite/host to its local host/parasite. Theory holds that under relatively strong parasite-induced selection, high specificity, limited migration and drift, parasites, with their shorter generation times, are adapted to their most locally common host genotype [8–11]. A standard method to test for LA is to conduct a reciprocal transplant, where either parasites or hosts are transplanted in the field [12]. If parasites are locally adapted, they are expected to be better able to infect and have higher fitness on local, *i*.*e* sympatric, than on foreign, *i*.*e* allopatric hosts [12, 13]. This has been supported empirically in some cases [14–18], but not in others, for instance when host gene flow exceeds that of the parasite or when parasites have long generation times [8, 19–21]. The extent of parasite generalism can also affect whether a parasite is adapted to its host, with some studies finding that LA depends on host breadth of the parasite [22] and others finding support for LA even among generalist parasites [21, 23].

Adaptation of malaria to its mosquito vectors has been demonstrated experimentally in a study of the human malaria parasite, *P*. *vivax*, adapted to its mosquito vectors, *Anopheles pseudopunctipennis* and *A*. *albimanus* [24]. Avian malaria and related parasites: *i*.*e*, *Plasmodium*, *Haemoproteus* and *Leucocytozoon* are a good candidate to study coevolution: they are both common and diverse, consisting of over 950 unique lineages worldwide [25–27]. Different lineages can also exert varying effects on host fitness [28–32]. In a famous case of avian malaria in Hawaii, where *Plasmodium relictum* was introduced in the 19^th^ century, malaria has been held responsible for the declines and extinction of several native bird species [33, 34]. Infection with malaria has also previously been associated with a deterioration in fitness-relevant host traits such as body mass [34, 35], haematocrit, *i*.*e* the proportion of red blood cells in the blood [36–38], oxidative stress [39] and fever in some [40] but not in all cases [36, 41, 42]. For instance, there is population specific allelic variation at MHC loci in house sparrows, *Passer domesticus*, infected with *P*. *relictum*, a generalist parasite, consistent with a pattern of diversifying selection and LA [43–45]. However, to truly determine whether generalist malaria parasites are locally adapted to their avian hosts experimental tests are needed.

We conducted one of the first, to our knowledge, LA studies of malaria parasites to their vertebrate hosts. The only previous study that has attempted this showed that haemogregarine blood parasites of the Canarian lizard, *Gallotia galloti*, were maladapted to their hosts [20]. We conducted a reciprocal transplant experiment in the wild, where we transplanted the host: juvenile and uninfected great tits, between our two study populations. Our two field sites in Switzerland differ in their dominant parasite lineage [46–48]. At one site, the lineage *Plasmodium relictum*, lineage SGS1 is by far the most common and at the other *Plasmodium polare*, SW2, and TURDUS1 co-occur. *P*. *relictum* infection has been linked to effects on host fitness [31] and *P*. *circumflexum* with effects on host survival in a study population of blue tits, *Cyanistes caeruleus* in the UK [32]. SGS1 and TURDUS1 are both generalist parasites and so by doing this experiment we can further explore the potential of generalist parasites to be locally adapted.

To quantify the extent of parasite local adaptation, we measured traits relating to the success of the infection as well as the ability to infect a host and at which intensity [17]. We also measured traits relating to the effects of infection on the host or parasite virulence, such as haematocrit, temperature and oxidative stress. These have been shown to be correlated to severity of infection and also to host fitness [35–37]. Empirical studies support that virulence is higher on sympatric than allopatric host as the parasite evolves to maximise its growth and fitness on a specific host genotype (e.g [14]) though it can also vary depending on which lineages are present, the relationship between virulence and transmission, the environmental conditions and spatial scale considered (reviewed in [49]). For malaria parasites to be adapted to their local hosts we therefore expect: i) infection rates to be higher on local (sympatric) versus transplanted (allopatric) hosts ii) local parasites to have a higher intensity on local than on foreign hosts if intensity is correlated to fitness iii) birds infected by local parasites to suffer more due to infection. Our set up also offers a unique opportunity to study the timing of a natural avian malaria infection and to gain much needed insight on the pathology of avian malaria infection in the wild.

## Materials and Methods

### Ethical statement

This experiment was approved by the Ethical Committee of the Vaud Canton veterinary authorities, licence number 1730.2. Birds were ringed under licence with the permit number F044-0799 of the Swiss Federal Office for the Environment.

### Experimental setup

The timing and duration of the experiment was planned in order to coincide with great tit reproduction, the introduction of juvenile uninfected birds into the populations and heightened vector activity in summer. We monitored great tits breeding in nest boxes at our two field sites: Dorigny, on the University of Lausanne campus (46°19’N; 6°49’E; alt. 400 m) and Monods, in a woodland surrounding a marsh (46°49’N; 6°49’E; alt. 680 m). Nests were checked regularly during April- May 2013, in order to establish the hatching date. Once the chicks had hatched, half a tablet used for insect repellent bracelets (Parakito, France) was fixed to the wooden part of the nest box. Every three days, nest boxes were sprayed with an insecticide mixture (PréButix Répulsive, Pierre Fabre, France) to deter vectors that could transmit avian malaria. Chicks were blood sampled when they were 14 days old to ensure that none were infected in the nest. The sample was collected using a heparinised microvette (Sarstedt®), and stored on ice. When the chicks were 17 days old, the two heaviest in the brood were taken back to indoor cages in a mosquito-free room where they were hand-raised (for sample size, see Table 1), as great tit nestlings rely on parental feeding for up to two weeks post fledging. The heaviest were chosen in order to ensure maximum survival in the lab. For the first day in captivity, chicks were force-fed a mix of beef heart and mealworms mixed with two commercially available food mixes complete with vitamins (canary mix, Holland Cova, Switzerland and a mix for hand-rearing young birds, Eric Schweizer, Switzerland). They were fed every hour for 13 hours a day (07:00–20:00). Every three hours they were given 100μl of diluted vitamins (Combex Multivitamin, Quiko). This regime continued until they achieved complete independence, characterised as being able to forage for meal worms and peck at the food mix alone. This took two weeks on average.

**Table 1:** Summary of sample sizes.

Release site	Site origin	Treatment	n birds at start	n birds at end (n_inf_)	n male	n female	n control at start (n_end_)
Dorigny	Dorigny	local	13	12 (4)	8	4	4 (4)
	Monods	foreign	19	16 (8)	10	6	5 (5)
	total	* *	32	28 *(12)*	18	10	9 (9)
Monods	Monods	local	18	15 (5)	10	5	5 (5)
	Dorigny	foreign	15	14 (8)	9	5	6 (5)
	total	* *	33	29 (13)	19	10	11 (10)

The number of birds at each release site according to site of origin, sex, number of controls (*ie* treated with Malarone) and number infected by the end of the experiment.

When several birds had reached independence at the same time, they were placed into the outdoor aviaries (1m x 1m x 2m, with a metal grid mesh size of 1cm x 1cm) situated close to the woodland edge at the two sites. Placing birds into the experiment therefore occurred on four dates (blocks), between June 13^th^, 2013 and July 5^th^ 2013. Birds were randomly assigned to each treatment group. Three birds were placed per aviary, provided with sunflower seeds, food mix and water containing vitamins *ad libitum*. As infection rates in some years are as high as 94% at our two field sites [46], we wanted to retain some uninfected birds in our setup. This was in order to compare site versus infection effects. To prevent infection, we randomly selected a portion of birds to be treated with a dose of commercially available Malarone® (GlaxoSmithKline), a mix of atovaquone/proguanil at a concentration of 1.08mg in 40 μl. We doubled the high dose used by Knowles et al. [31] as this has been shown in previous work from this study system to clear blood stages of malaria parasites in chronically infected birds [50]. Birds from the same nest were placed at different sites, and were randomly allocated to aviary and treatment group (Malarone/PBS, local/foreign). At the end of the experiments birds were released in the wild.

### Bird measurements

Blood samples and bird measurements were taken both before placing the birds outside and at time points throughout the course of the experiment (days 14, 21, 35, 49, 63 and 77). At these time points birds were also given either malarone or PBS. The following measurements were taken:

1. **Morphometric:** tarsus length was measured using electronic metal calipers, correct to the nearest 0.01mm. Birds were also weighed on an electronic balance to the nearest 0.1 g.
2. **Cloacal body temperature:** was measured using a digital thermometer to the nearest 0.1°C (Sensortrek- BAT-12).
3. **Oxidative stress:** 16μl of blood was immediately pipetted into 584 μl of KRL buffer (Kirial international, Laboratoires Spiral S.A., Dijon, France), a physiological buffer adjusted to bird osmolarity. This was used for the determination of oxidative stress level, taken as red cell membrane resistance to free radical attack, and the time needed to haemolyse half the red blood cells, measured using a spectrophotometer at 540nm [51].
4. **Haematocrit:** a drop of blood was collected in a haematocrit capillary tube (Assistent). Capillaries were placed in a capillary microcentrifuge and spun for 10 mins at 14 000 rpm.

### Molecular analyses

#### Parasite identification and quantification

After collection, blood samples were centrifuged for 10 min at 15 000 rcf at 4°C. Red blood cells and plasma were split and stored separately at -20°C, for subsequent analysis.

DNA was extracted from the red blood cells using the spin column method of the DNeasy blood and tissue extraction kit (Qiagen, CA) according to the manufacturer’s protocol. Avian genomic DNA concentration was quantified using the Qubit® Fluorometer (Life Technologies), according to the protocol for small samples, and diluted to a working concentration of 10ng/μl. A nested PCR was performed with HaemF and HaemR2 primer pairs to detect *Plasmodium*, and HaemFL and HaemR2L primer pairs to detect *Leucocytozoon*, a related clade of parasites [52]. PCRs were run in duplicate, with one negative control (water) for every 15 samples. One positive control was included for every 30 samples. Infection was confirmed by running 5 μl of the secondary PCR products on an agarose gel and where positive, the products were sent to Microsynth (Switzerland) for purification and sequencing in both directions using HaemF and HaemR2 primer pairs. Sequences were aligned using the software MEGA 5.2.2 [53] and identified using a local BLAST search against the MalAvi database [54].

For *Plasmodium* positive samples, parasite quantification was performed as described in Christe et al. [46] using a parasite cytochrome b TaqMan probe (CY3-CYTb-BHQ2: 5’-CCTTTAGGGTATGATACAGC-3’) and a host 18s rRNA probe (FAM-18S-BHQ1: 5’-AACCTCGAGCCGATCGCACG-3’). Host and parasite plates were run separately. For the standard curve, two-fold serial dilutions starting from 10ng/μl of bird genomic DNA, of a reference sample that was known to be heavily infected, were run along with the test samples and negative controls (water). As a second control, to ensure our diagnostic nested PCR did not miss some infections we conducted the qPCR reaction on 20 negative samples. All samples and controls were run in duplicate. Parasitaemia was calculated as follows:

$$\alpha =10^{\frac{I-y}{m}}$$

The DNA concentration (α) is calculated with the intercept (I) and the slope of the regression line of the standard curve (m) and the sample’s CT (y). The parasitaemia (r) is given by the ratio of the parasite DNA concentration on the host DNA concentration:

$$r=\frac{\alpha \;parasite}{\alpha \;host}$$

In order to normalise the distribution, r was log_10_ transformed. As a consequence, we obtained a range of parasitaemia values ranging from -4.91 to -0.28 (unitless). The range was negative as the reference sample used for the standard curve came from a more heavily infected bird from a previous study.

#### Molecular sexing

The birds’ sex was determined using a PCR reaction with the primers SPIN 375 (forward), SPINZ 12- rev and SPINW 12-rev, that target the sex chromosomes of birds [55]. Reactions were performed in a final volume of 25μl with 1 x Qiagen PCR buffer, 3.5mM of MgCl_2_, 0.2mM dNTP mix, 0.5μM of SPIN375, 0.25μM of SPIN-Z and 0.25μM of SPIN-W and 0.2 units of Taq. The PCRs had the following conditions: 94°C for 30s, 50°C for 30s, 72°C for 30s, for 49 cycles, followed by a 94°C incubation for 2 mins before and a 72°C for 5 mins at the end of the reaction.

### Statistical analyses

We first tested for a difference in sex ratio at the two sites using a contingency table analysis (GLM, with Poisson errors). All other response variables were analysed using either linear or generalised linear mixed effects models (GLMM) as implemented in the “lme4” package [56] in R v.3.0 [57]. In all models, we included random effect terms for aviary, nest of origin and temporal block. In cases where a random effect explained zero variance, the effect was removed, provided it did not lead to an increase in model AIC (for model formulae see S1 File).

#### Parasite variables

We explored the effect of treatment on infection, parasitaemia and the timing of infection.

As most infections occurred towards the end of the experimental period, we had few individuals with multiple (*i*.*e*. > four) measurements. Therefore, we were not able to analyse the infection dynamics using a time- series analysis. Instead, we analysed i) the probability of infection during the course of the experiment, *i*.*e* whether a host got infected or not, ii) peak parasite intensity detected for each individual, as this can be related to both virulence and fitness and iii) final parasite intensity: *i*.*e*. by the end of our experimental period, which could be considered an indicator of the cumulative cost of infection. To test for LA, we included a “site of origin x release site” interaction term in each of our analyses. We also included terms for treatment (Malarone or PBS) and repeated the analysis for non-treated birds separately. In the case of maximum parasite intensity, which varied depending on date, we also included date (coded as days since the 1^st^ of July) as a covariate.

#### Variables related to the effects of infection on the host

We investigated whether infection during the experiment (presence/absence) was related to host physiological variables: body condition measured as the scaled mass index (SMI), haematocrit, temperature and red blood cell membrane resistance, an indicator of previous oxidative damage. SMI was chosen because it has been shown to perform better than standard OLS methods of regressions of log body size on length [58].We analysed the measurements taken at the two time points we considered above: i) peak parasite intensity detected and ii) parasite intensity by the end of the experiment. As the timing of maximum parasite intensity varied between individuals and sites and because we wanted to compare uninfected with infected birds at each site, we first established the median date at which birds reached their highest infection intensity at each site separately. We then analysed parameters for uninfected individuals, measured closest to this median date.

To account for the fact that individuals may vary in their baseline value for a trait (e.g SMI), we first plotted the relationship between the traits at the start and at the measured time point. If the two were correlated, we included the starting value as a covariate in order to correct for it. We also checked whether the trait correlated with sampling date and if so, date was also included as a covariate. An “origin x release site” interaction could also arise because individuals respond differently depending on their site of origin (due to similar genetics) or release site (a common environmental component at the release site). In this case, we would expect to see similar effects on host variables in uninfected and infected birds. Therefore, we expect LA to be detected as an “infection x site of origin x release site” interaction. Though potentially important, we could not include interactions between sex and infection, as the number of infected females at each site was low. We therefore included sex as a covariate, alongside treatment (Malarone or PBS).

We repeated the above analyses with infected individuals only and logr (parasite intensity measured either at the peak or at the end of the experiment) as a covariate and the interaction of interest (“origin x release site”). For a full list of models see S1 File.

#### Model averaging

The global model from GLMM or LMM was used in an information theoretic model averaging procedure as outlined in Grueber et al. [59] implemented in the package MuMIn [60]. Information theoretic approaches are gaining popularity in the study of ecology and evolution [61, 62] and can also be useful when interpreting experimental data [63]. This could be especially applicable in situations where the best method of interpolating significance is still a contentious issue [64].

**Fig 1:**
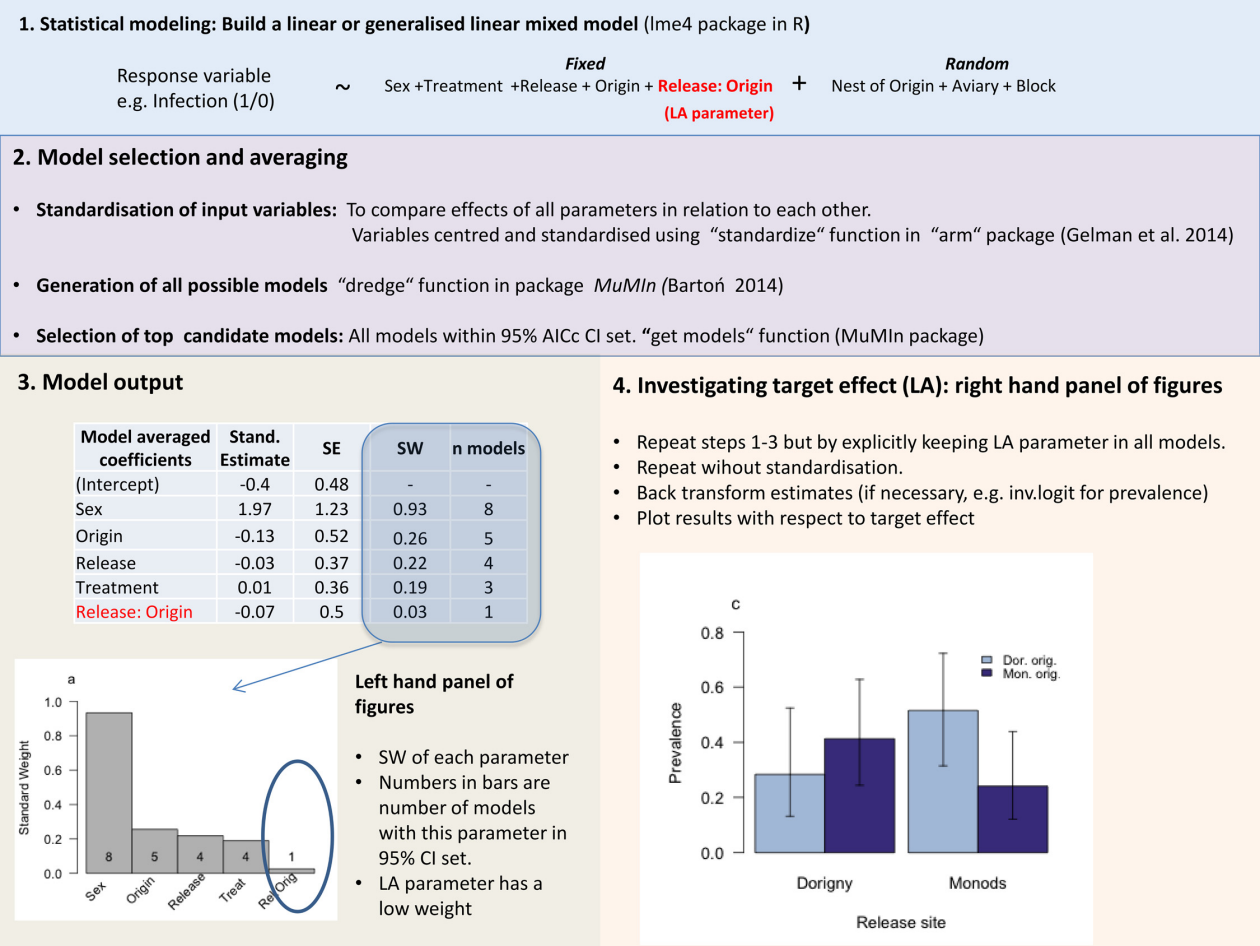
A step-by-step guide to the statistical modelling and the model selection and averaging procedure implemented (modified from [59]).

Briefly, after running the LMM/GLMM a set of candidate models were produced with all fixed effects and interactions of interest, e.g the LA parameter. We respected the principle of marginality, meaning that main effects had to be included in a model if the interaction was present. In order to directly compare the effect sizes of each of the parameter estimates, we first standardised the input variables to a mean of zero and a SD of 0.5, using the function “stdz.mod” in the R package “arm” [65]; this standardises categorical variables [66]. We constrained our model sets to include all covariates that we wanted to correct for (e.g starting value of host parameter and/or date). The relative likelihood of each model was then estimated using normalised ΔAICc weights (AIC corrected for small sample sizes), and the top set of models was selected as those within the 95% confidence interval of summed weights [63]. It is worth noting that this method does not allow for direct hypothesis testing and therefore does not report p-values. Instead, it calculates the standard weight [67] (also known as relative importance in other publications e.g. [59]), based on how many times it occurs in each of the top set of candidate models and the relative weight of the models in which it occurs in [63]. For a step-by-step implementation of the statistical methods and the model averaging procedure, see Fig 1.

## Results

All birds were uninfected at the start of our experiment, and none tested positive for *Leucocytozoon* spp. at any time-point. 57 out of 65 birds placed into the experiment survived and were measured at the end (Table 1). Six of the eight dead birds were recovered intact, dissected and their livers analysed: two were found to be PCR positive for *Plasmodium* spp., though these birds were not included in the analysis. There were more males than females in our setup (Likelihood Ratio Test: χ^2^ = 5.43, d.f = 1, p = 0.02), though this was not biased per site (Table 1). The timing of infection during the season was best predicted by release site and treatment (SW = 0.62/0.63). Birds in Dorigny got infected on average two weeks earlier than in Monods (mean date Dorigny = 23^rd^ August 2013). Despite the treatment, malarone treated birds got infected at the same rate as control birds, but on average ten days later (6^th^ September 2013 versus 27^th^ August 2013). There were also no differences in parasite intensity among treated and non-treated birds (SW = 0.22). The prevalence was 45% at both of the sites, yet the lineages identified differed: TURDUS1, *Plasmodium circumflexum* spp. was the only parasite found in the Monods birds and SGS1, *P*. *relictum* was the only one found in Dorigny. None of the 20 birds that were diagnosed as uninfected amplified in the qPCR reaction.

### Parasite variables

**Fig 2:**
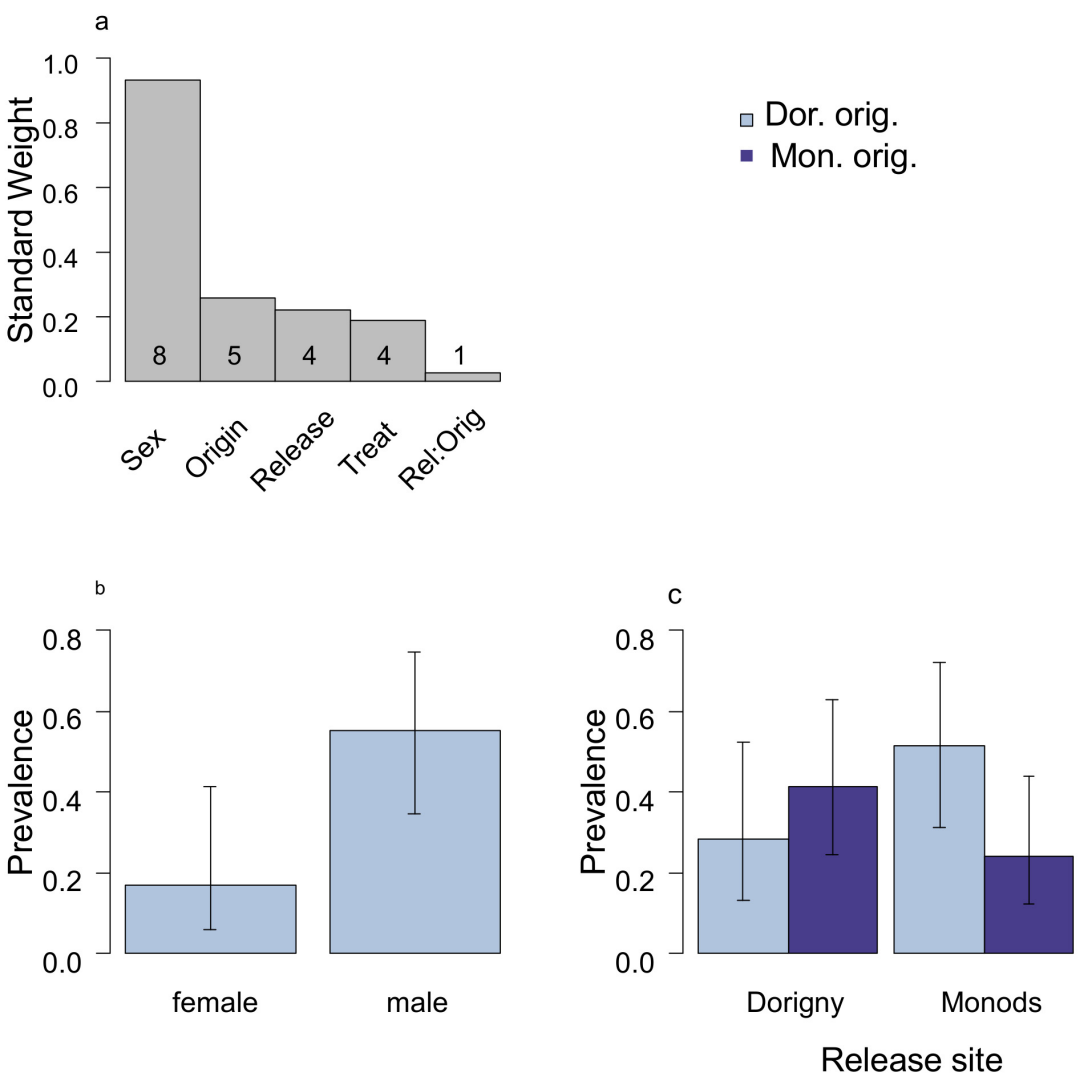
The factors that affect the likelihood of infection by the end of the experimental period. **a)** The standard weight of parameters in the 95% confidence interval model set and the number of candidate models that they occur in after model averaging. b) The analysis repeated on the original scale and predicted prevalence ± 1 se for males and females. c) Exploring the direction of the target effect, LA, by explicitly keeping the LA parameter in all models. Under a scenario of parasite adaptation, we expect parasites to be more infective on local hosts. Prevalence is plotted on the original scale. For further details of the method see Fig 1.

**Fig 3:**
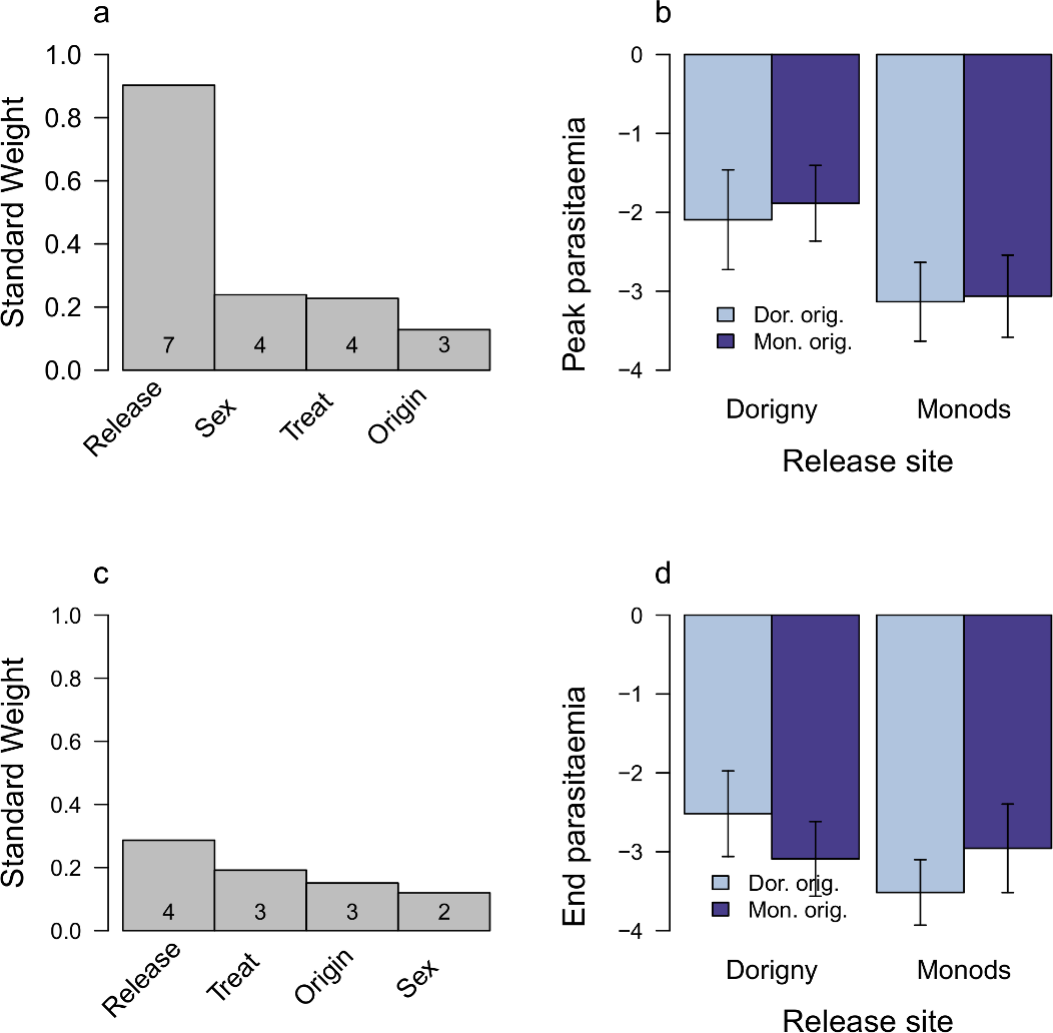
The factors that affect maximum parasite intensity attained (a-b) and intensity by the end of the experiment (c-d). The left hand panels (a and c) give the standard weight of the predictor parameters in the 95% confidence interval model set and the number of candidate models that they occur in. In the right hand panels, the direction of the LA parameter (Origin x Release site) is explored by explicitly keeping the LA parameter in all models. Predicted parasite intensity is plotted on the original scale. Note that parasite intensity is expressed in units relative to a reference sample from a previous study, which had a higher intensity than the ones in this study. Therefore, the values are negative and those closer to zero indicate a higher parasite intensities. For further details of the method see Fig 1. Under a scenario of parasite adaptation, local parasites will have a higher intensity on local hosts.

Males were twice more likely to get infected than females (Fig 2B). The LA parameter (release site x site origin) had a low weight. The direction of the effect however, was consistent with local hosts being less likely to get infected (Fig 2C). This was also the case when considering treated birds only (Tables A-B in S2 File). Release site best predicted maximum parasite intensity recorded at each site (Fig 3A and 3B, Table C in S2 File), yet this difference was less pronounced by the end of the experiment (Fig 3C and 3D, Table E in S2 File). The pattern was consistent when repeating analyses without the malarone-treated birds (Tables D and F in S2 File).

### Effects of infection on the host

**Fig 4:**
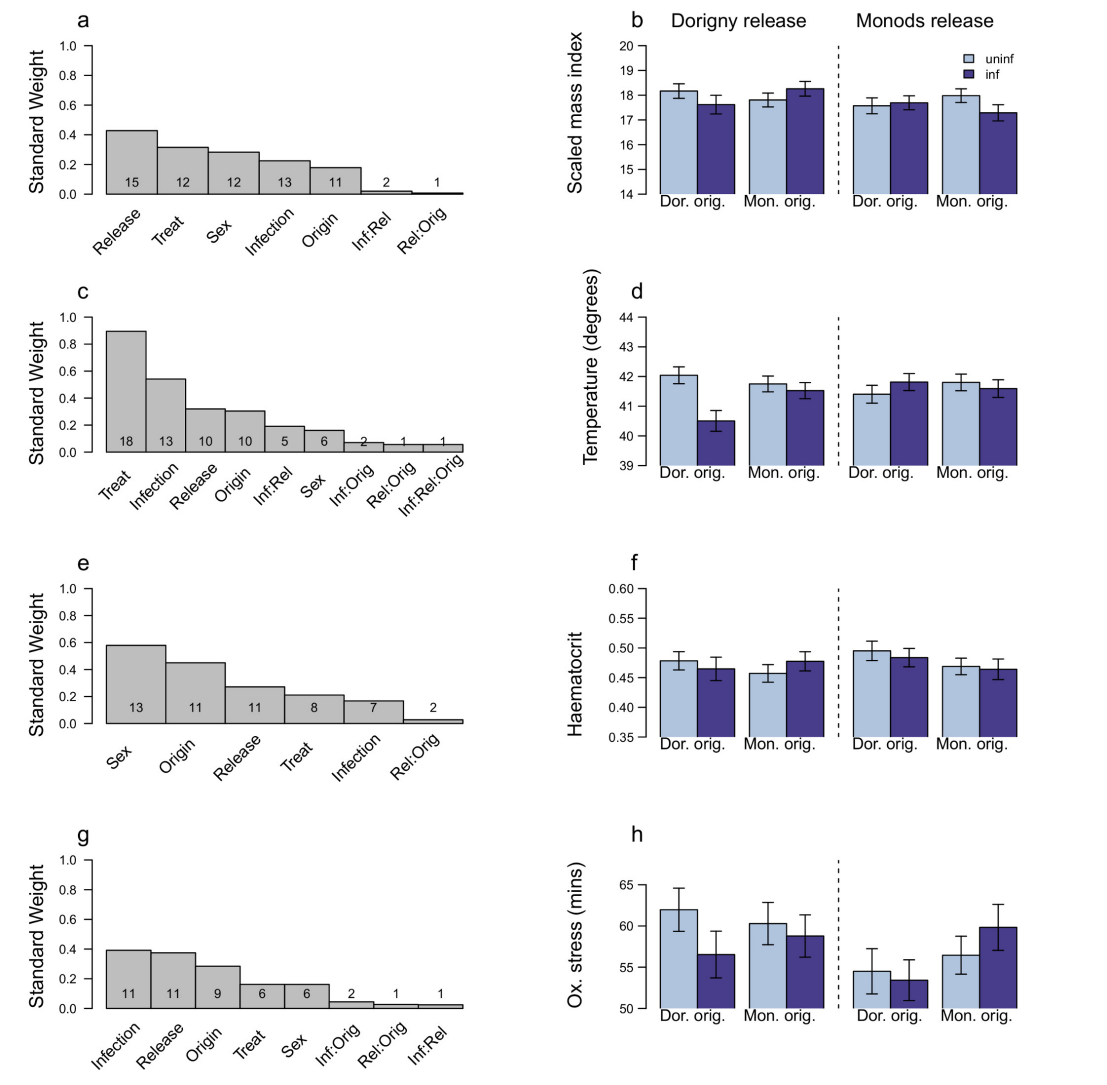
The factors that affect variables related to host pathology at the infection peak. a- b) SMI: standardised mass index; c- d) temperature; e- f) haematocrit; g- h) oxidative stress: membrane resistance (time taken to haemolyse half of red blood cells when faced with free radical attack). The left hand panels (a, c, e, g) show the standard weight of the predictor parameters affecting the host response variables and the number of candidate models in the 95% confidence interval model set that they occur in. Note that the interaction term indicative of host LA (Infection x Origin x Release site) is not always present. In the right hand panels (b, d, f, h) the LA effect (Infection x Origin x Release site) is explored and in these models, the LA term is explicitly retained. The predicted model averaged parameter estimates ± 1 se of each response variable is plotted on the original scale. Note that parameters that were included as covariates were set as “fixed”, *i*.*e*, the SW = 1, and they are therefore not shown in the plots. For full model formulae and results see tables in S1 File and S3 File. For further details of the method see Fig 1. The prediction, under a scenario of parasite adaptation, is that local birds will suffer more and have lower SMI, temperature, haematocrit and membrane resistance.

**Fig 5:**
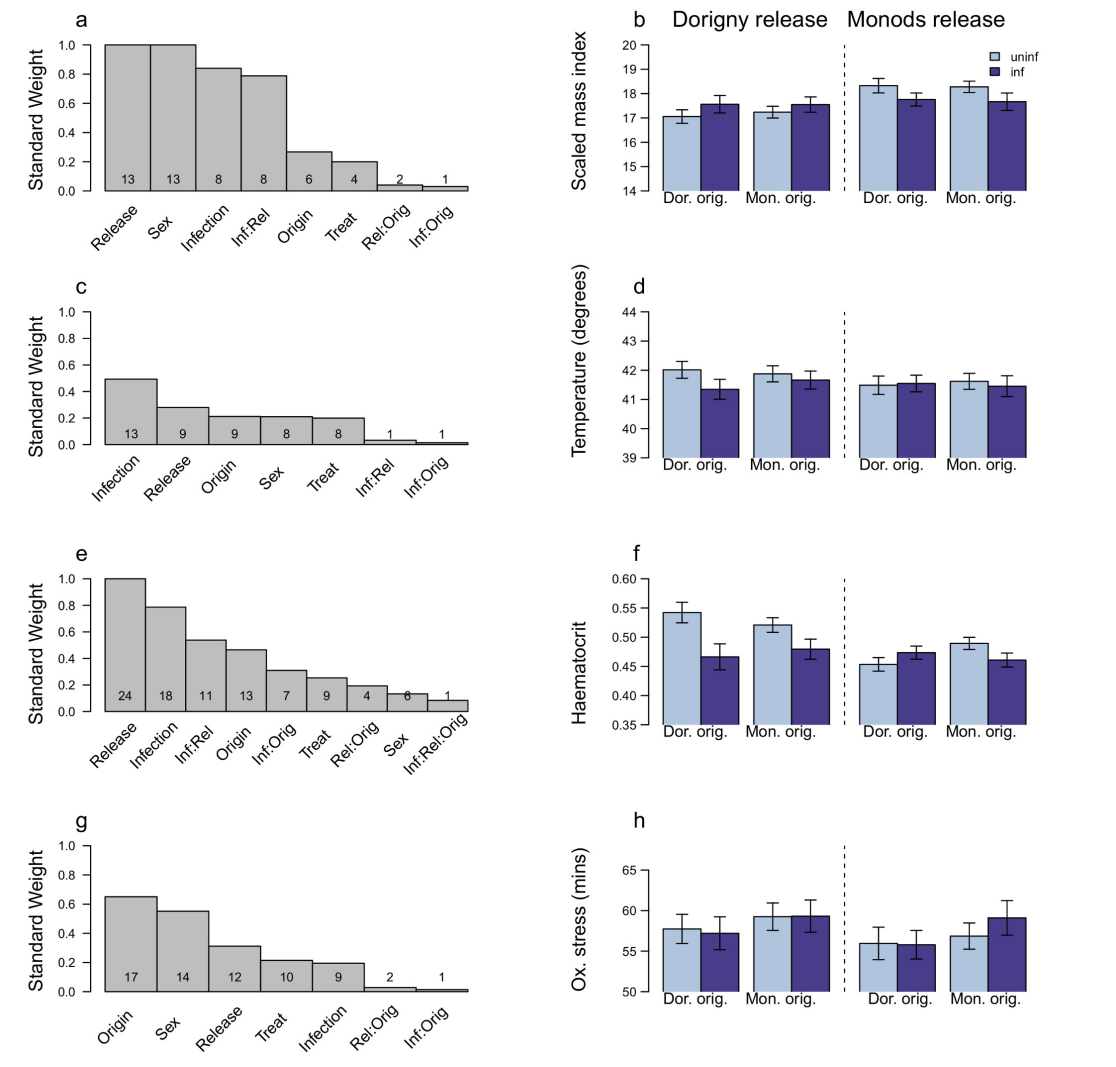
The factors that affect variables related to host pathology by the end of the experiment. a- b) SMI: standardised mass index; c- d) temperature; e- f) haematocrit; g- h) oxidative stress: membrane resistance (time taken to haemolyse half of red blood cells when faced with to free radical attack). The left hand panels (a, c, e, g) show the standard weight of the predictor variables affecting the host response variables and the number of candidate models in the 95% confidence interval model set that they occur. Note that the interaction term indicative of host LA (Infection x Origin x Release site) is not always present. In the right hand panels (b, d, f, h) the LA effect is explored (Infection x Origin x Release site) and in these models, the LA term is explicitly retained. The predicted model averaged parameter estimates ± 1 se of each response variable is plotted on the original scale. For full model formulae and results see tables in S1 File and S3 File. For further details of the method see Fig 1. The prediction, under a scenario of parasite adaptation, is that local birds will suffer more and have lower SMI, temperature (given previous studies have shown that infected birds can have a lower temperature), haematocrit and membrane resistance.

Infection had some effects on physiological parameters, measured either at the peak of infection or at the end of the experiment. The LA parameter (i.e, infection x origin x release site) was retained in only few of the analyses (Figs 4 and 5, Tables in S3–S6 Files).

When birds suffered the highest infection intensity, there was no consistent effect of infection on body condition, haematocrit or oxidative stress level (Fig 4A and 4B, Fig 4E–4H, Tables A, C-D in S3 File). Infection predicted temperature in about half the models (Fig 4C, Table B in S3 File). In Dorigny, infected birds of local origin had lower temperature than uninfected ones (Fig 4D). Explicitly including parasite intensity in the models was not informative for any of the host parameters measured at the infection peak (Tables in S4 File).

**Fig 6:**
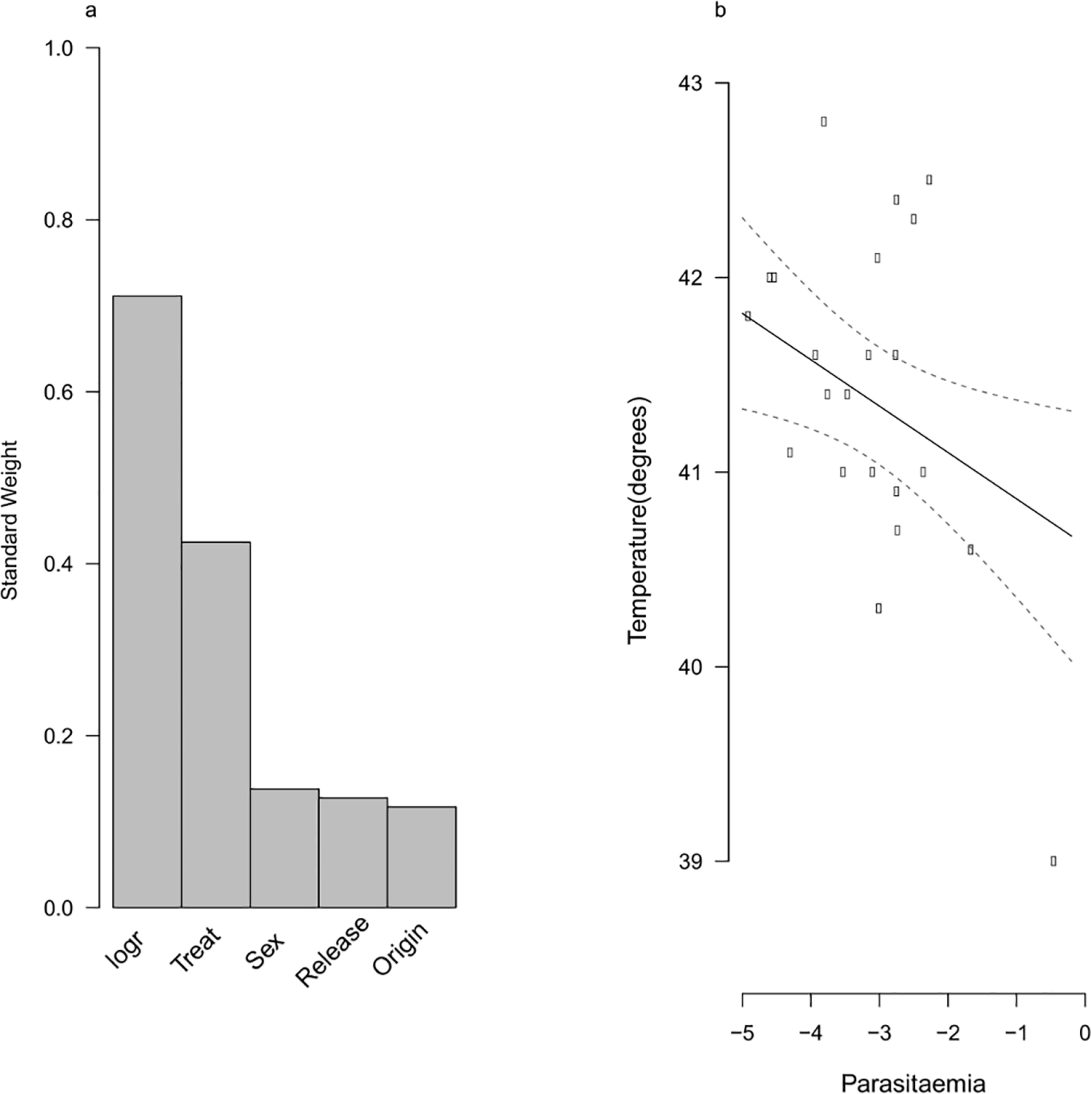
The relationship between temperature and infection intensity. **a)** The standard weight of variables affecting temperature measured at the end of the experiment and the number of candidate models in the 95% CI model set measured at the end of the experiment with parasite intensity (logr) fitted explicitly. b) Predicted model averaged estimates of temperature (degrees) in relation to parasite intensity (logr).

By the end of the experimental period, infection was not a good predictor of body condition, temperature or oxidative stress (Fig 5A–5H, Table A,C-D in S5 File). Haematocrit was affected by release site, infection and an interaction between the two (Fig 5E, Table C in S5 File). Overall, birds released in Monods had lower haematocrit than those released in Dorigny, however infected birds in Dorigny had lower haematocrit than uninfected ones (Fig 5F). Parasite intensity best predicted temperature (SW = 0.71) and there was a negative relationship between parasite intensity measured at the end of the experiment and body temperature (Fig 6, Table B in S6 File). Explicitly accounting for parasite intensity resulted in similar results as found previously for the other variables (Tables A, C-D in S6 File).

## Discussion

Experimental tests of LA are often complicated to perform in wild vertebrate systems. Here, we present one of the first, to our knowledge, reciprocal transplant experiments using the avian malaria—great tit system. This experimental set up also allowed us to monitor the timing and course of natural avian malaria infection in the wild. We found marked sex differences in terms of infection success, with males more likely to get infected than females. We also report differences with respect to the infection intensity of parasites at the two sites and further effects of primary infection on host pathology.

### Potential for LA

We find no evidence for LA of malaria parasites to their great tit hosts for any of the traits that we investigated. When considering whether birds get infected or not, it is worth noting that the direction of the LA effect is consistent with local hosts being less likely to get infected, which is maladaptive for the parasite. This could also indicate that hosts are better able to resist local parasites (host adaptation). Maladaptation has previously been found in a lizard- haemogregarian blood parasite system [20], a situation that can arise when migration rates of the host are intermediate yet higher than those of the parasite [8]. Indeed, both lizards and great tits undergo a post-juvenile dispersal phase and the latter, though mostly sedentary, disperse several hundred metres–a few kilometres [68]. Whether there is a general tendency for maladaptation remains to be elucidated.

A lack of LA can also occur for other reasons; for example high dispersal can result in a homogenisation of host resistance alleles across populations, thereby limiting the response to selection [69]. Ringing recoveries have revealed that great tits are able to occasionally travel long distances of between tens to hundreds of kilometres [70]. Though we have had no ringing recoveries between our two sites, we cannot exclude the possibility of dispersal between the two.

Parasite specificity to the host and vector can also affect the strength of LA [10], the idea being that specialised parasites evolve adaptations to best infect and survive in their target host and vector species. *P*. *relictum* (SGS1) is a cosmopolitan parasite that occurs worldwide and that has been isolated from 100 host species spanning eleven orders. *P*. *circumflexum* (TURDUS1) is also a generalist and has been found in 28 hosts, but is restricted to Europe and Africa [54]. However, even generalist parasites have the potential to be locally adapted or maladapted. Host races of the generalist tick, *Ixodes uriae* are locally adapted to its host, the black-legged kittiwake, *Rissa tridactyla* [23] and the hen flea, *Ceratophyllus gallinae* is maladapted to some great tit populations [21]. Adaptive specialisation of generalist parasites on certain hosts could explain this. In the tick-kittiwake example, the engorgement success of nymphs obtained from the focal kittiwake host was higher than that of nymphs that had been transplanted from another sympatric seabird species [71]. It is therefore clear that the position of the parasite in the generalist-specialist continuum and potential for cryptic variation in host preference of generalist parasites is important to consider.

In some situations, there can be LA of the parasite to the vector, as in the case of the human malaria parasite, *P*. *vivax*, which is better able to infect its sympatric *Anopheles* spp. [24]. Alternatively, the vector itself can be highly generalist with broad feeding preferences. Recent molecular studies have isolated the same lineages of *Plasmodium* parasites from multiple mosquito species [72, 73]. At our Dorigny site, we have identified *Culex pipiens* as the most likely vector of *P*. *relictum* (SGS1) [47]. Studies in Japan have shown the broad feeding preferences of *Culex* mosquitoes [74, 75] and SGS1 has been found in seven mosquito species (MalAvi, 2015). *P*. *circumflexum* (TURDUS1) however, is mainly thought to be vectored by *Culiseta* spp (Valkiūnas 2005). The two sites therefore differ in their insect and host communities [47], and so the transmission potential and nature of coevolutionary interaction is also likely to vary. Ultimately, the strength of the divergent selection pressures exerted by multiple hosts/vectors, will determine the outcome of LA to a particular host/vector species [22]. In summary, host migration and low parasite specificity to either the vector or the host could be why we do not detect LA. Controlled infection experiments in a laboratory setting or carefully designed field experiments with replicates of paired parasite-host populations where there is knowledge about the regional host, parasite and vector ecology could shed light on some of these effects.

### Sex-biased infection

One of the most marked results was a sex-biased pattern of infection; males at both sites were more likely to get infected than females. This effect has been found frequently among adult vertebrates and has been attributed to differences in ecology, behaviour and physiology between the sexes (e.g [76] and reviewed in [77, 78] but see [79]). This effect has rarely been investigated among juveniles, though one study showed that male great tit nestlings suffer a greater reduction in growth rate due to ectoparasite infestation [80]. It is possible that the antagonistic role of testosterone on immune defense could leave males more susceptible to infection, particularly in sexually dimorphic species where they can also invest in costly ornaments [81–83]. The higher susceptibility to infection could be explained by a peak of testosterone activity in juvenile males in September [84]. Alternatively, males which are larger and expire more CO_2_ may be more attractive to vectors. The two may also interact and testosterone could also affects host metabolic rate [85], thereby influencing host attractiveness to vectors. Though the mechanism underlying this effect remains to be elucidated, it is tantalising that such differences are already present at the start of infection.

An added advantage of our experimental set up was that it allowed us to observe the timing of the natural infection. Previously, it was thought that most transmission occurs in early summer when adults experience a relapse in infection and there is a release of immunologically naïve juveniles in the population [86]. A study on domestic turkeys placed in the habitat of wild turkeys in Florida showed that transmission depended on the lineage, vector availability and season [87]. We find that surprisingly, infection occurred relatively late in the season, about six weeks after the birds being placed in the outdoor aviaries. This suggests that primary infection, at least at these two sites, does not occur when the chicks are still in their nest or even as soon as they fledge and may be due to the later emergence of vectors [88].

### Parasite effects on host pathology

The two parasite species found at our two sites varied in intensity, a trait that can be linked to virulence [89, 90] but see [91]. Parasite intensity was higher in Dorigny where *P*. *relictum* is the main circulating strain, suggesting that this parasite could be more virulent than *P*. *circumflexum*. Alternatively, it can also be a site-specific effect of strain and local environment that affects the disease severity. The virulence of *P*. *relictum* is supported by experimental infections [36, 92]. A recent study of blue tits in the UK also predicted that *P*. *relictum*, with its broader dispersal capacity, is also likely to be more virulent than *P*. *circumflexum* [93]. In Dorigny, locally originating infected birds had a low body temperature, consistent with results from experimentally infected canaries [41], but see [36]. Interestingly, this effect was not pronounced among uninfected or Monods origin birds. Birds naturally have a high body temperature (range 39°C- 43°C) and it could be that a temperature reduction is beneficial to the development of the parasite. Our finding that temperature decreases with infection intensity as measured at the end of the experiment is consistent with this hypothesis. Infected birds in Dorigny also had a lower haematocrit, confirming that infection causes anaemia [92]. Taken together, it appears that there are some site and/or lineage specific effects of infection.

## Conclusions

We found no evidence for LA of malaria parasites to their host in the wild. However, this study reveals that such experiments are in fact possible with wild vertebrates and we provide an experimental framework by which one can attempt them. Our results shed light on the timing of infection at our two sites which was later than previously anticipated from the literature. In addition, we found effects on traits relevant to host fitness and pathology, indicating that *Plasmodium* spp. harm their hosts and therefore have the potential to act as selective agents. We also report a sex-biased infection rate in young birds and propose that further studies should explore the mechanisms underlying this effect. It is possible that such a strong sex-difference could have implications on transmission dynamics and on the evolution of virulence [94].

## Supporting Information

S1 File All the statistical models in the R syntax. (DOCX)

S2 File Models of parasite variables. **Table A** infectivity: when a bird is infected or not, **Table B:** infectivity in non-malarone treated birds. **Table C:** Peak parasitaemia. **Table D:** Peak parasitaemia in non-malarone treated birds.
(DOCX)

S3 File Models of host variables at the infection peak Table A SMI: standardized mass index, Table B: temperature. Table C: haematocrit. Table D: oxidative stress measured as membrane resistance. (DOCX)

S4 File Models of host variables with parasite intensity at the infection peak. **Table A** SMI: standardized mass index, **Table B:** temperature. **Table C:** haematocrit. **Table D:** oxidative stress measured as membrane resistance.
(DOCX)

S5 File Models of host variables at the end of the experiment. **Table A** SMI: standardized mass index, **Table B:** temperature. **Table C**: haematocrit. **Table D**: oxidative stress measured as membrane resistance.
(DOCX)

S6 File Models of host variables with parasite intensity at the end of the experiment. **Table A** SMI: standardized mass index, **Table B:** temperature. **Table C**: haematocrit. **Table D**: oxidative stress measured as membrane resistance.
(DOCX)
