# Supplementary material for: Testing Local Adaptation in a Natural Great Tit-Malaria System: An Experimental Approach
Source: PLoS One. 2015 Nov 10;10(11):e0141391. doi: 10.1371/journal.pone.0141391 (PMC4640884; doi:10.1371/journal.pone.0141391)
Supplement: S1 File — (DOCX) [file pone.0141391.s001.docx]

**Table S1. All the models used in the R syntax.** For all subsequent tables, SMI= Standardized Mass Index (an indicator of body condition)

| **Model** | **R syntax** |
| --- | --- |
| ***Effects of treatment*** |  |
| Date of infection | lmer(Date~Release site*Site origin+Sex+ Treatment+  (1\|Nest)+(1\|Aviary)+(1\|Block)) |
| Probability of infection | glmer(Infection~Release site*Site origin+Sex+ Treatment+  (1\|Nest), family="binomial") |
| Maximum parasitaemia | lmer(logr~Release site*Site origin+Sex+ Treatment+  (1\|Nest)) |
|  |  |
| ***Parasite variables*** |  |
| Infectivity | glmer(Infection~Site origin*Release site+ Treatment+Sex+  (1\|Nest)+(1\|Aviary)+(1\|Block), family="binomial") |
| Maximum parasitaemia | lmer(logr~Sex+Site origin*Release site+ Treatment+Date +  (1\|Nest) |
| Parasitaemia at end |  |
| of experimental period | lmer(logr~Site origin*Release site+Sex+Treat +(1\|Aviary)) |
|  |  |
| ***Host variables at peak of infection*** | |
| SMI | lmer(SMI max~Infection*Release site*Site origin+Sex+ Treatment+  Date+SMI 14+(1\|Aviary)+(1\|Nest)+(1\|Block)) |
| Temperature | lmer(Temp max~Infection*Release site*Site origin+Sex+ Treatment+  Date+(1\|Aviary)+(1\|Nest)) |
| Haematocrit | lmer(Haem max~Infection*Release site*Site origin+Sex+Haem 14+  Treatment+(1\|Aviary)) |
| Oxidative stress | lmer(tdemi max~Infection*Release site*Site origin+Sex+ Treatment+  (1\|Aviary)+(1\|Block) |
|  |  |
| ***Host variables at the end of the experimental period*** | |
| SMI | lmer(SMI end~Infection*Release site*Site origin+Sex+ Treatment+  SMI 14+(1\|Block) |
| Temperature | lmer(Temp end~Infection*Release site*Site origin+Sex+ Treatment+(1\|Aviary) |
| Haematocrit | lmer(Haem end~Infection*Release site*Site origin+Sex+Haem 14+ Treatment+(1\|Aviary)+(1\|Nest) |
| Oxidative stress | lmer(tdemi sept~Infection*Release site*Site origin+Sex+ Treatment+(1\|Aviary)+(1\|Block) |
|  |  |
| ***Explicitly including parasitaemia (logr) at peak*** | |
| SMI | lmer(SMI max~SMI 14+Release site*Site origin+logr+Sex+ Treatment+Date+(1\|Nest)+(1\|Block) |
| Temperature | lmer(Temp max~Release site*Site origin+logr+Sex+ Treatment+  Date+(1\|Aviary)+(1\|Nest) |
| Haematocrit | lmer(Haem max~Release site*Site origin+logr+Sex+ Treatment+  Date+(1\|Aviary)+(1\|Nest)+(1\|Block) |
| Oxidative stress | lmer(tdemi max~Release site*Site origin+logr+Sex+ Treatment+ Date+(1\|Aviary)+(1\|Block) |
|  |  |
| ***Host variables(parasitaemia at end of experiment as covariate)*** | |
| SMI | lmer(SMI end~SMI 14+Release site*Site origin+logr+Sex+ Treatment+(1\|Nest)+(1\|Block) |
| Temperature | lmer(Temp end~Release site*Site origin+logr+Sex+ Treatment+(1\|Aviary) |
| Haematocrit | lmer(Haem end~Release site*Site origin+logr+Sex+ Treatment+(1\|Nest) |
| Oxidative stress | lmer(Ox.stress~Release site*Site origin+logr+Sex+Treat+(1\|Aviary) |
