# Supplementary material for: Testing Local Adaptation in a Natural Great Tit-Malaria System: An Experimental Approach
Source: PLoS One. 2015 Nov 10;10(11):e0141391. doi: 10.1371/journal.pone.0141391 (PMC4640884; doi:10.1371/journal.pone.0141391)
Supplement: S2 File — Table A infectivity: when a bird is infected or not, Table B: infectivity in non-malarone treated birds. Table C: Peak parasitaemia. Table D: Peak parasitaemia in non-malarone treated birds. (DOCX) [file pone.0141391.s002.docx]

| **File S2. Models of parasite variables** | | | | | | | | | | | | | | | | |  |  |  |
| --- | --- | --- | --- | --- | --- | --- | --- | --- | --- | --- | --- | --- | --- | --- | --- | --- | --- | --- | --- |
| **A Infectivity** | |  | | |  | | | |  | | |  | | |  | |  |  |  |
| *Component models* | | *df* | | | *logLik* | | | | *AICc* | | | *Delta* | | | *Weight* | |  |  |  |
| Sex | | 3 | | | -34.96 | | | | 76.38 | | | 0 | | | 0.39 | |  |  |  |
| Sex+ Origin | | 4 | | | -34.82 | | | | 78.42 | | | 2.04 | | | 0.14 | |  |  |  |
| Release+ Sex | | 4 | | | -34.94 | | | | 78.67 | | | 2.3 | | | 0.12 | |  |  |  |
| Sex + Treat | | 4 | | | -34.96 | | | | 78.7 | | | 2.32 | | | 0.12 | |  |  |  |
| Release + Origin + Treat | | 5 | | | -34.8 | | | | 80.79 | | | 4.42 | | | 0.04 | |  |  |  |
| Intercept | | 2 | | | -38.29 | | | | 80.81 | | | 4.44 | | | 0.04 | |  |  |  |
| Sex + Origin +Treat | | 5 | | | -34.81 | | | | 80.82 | | | 4.45 | | | 0.04 | |  |  |  |
| Release + Sex + Treat | | 5 | | | -34.94 | | | | 81.08 | | | 4.71 | | | 0.04 | |  |  |  |
| Release + Sex+Origin+ Rel:Orig | | 6 | | | -33.8 | | | | 81.31 | | | 4.93 | | | 0.03 | |  |  |  |
| Origin | | 3 | | | -38.17 | | | | 82.8 | | | 6.43 | | | 0.02 | |  |  |  |
| Treat | | 3 | | | -38.23 | | | | 82.93 | | | 6.55 | | | 0.01 | |  |  |  |
|  | |  | | |  | | | |  | | |  | | |  | |  |  |  |
| *Model averaged coefficients* | | Estimate | | | SE | | | |  | | |  | | |  | |  |  |  |
| (Intercept) | | -0.40 | | | 0.48 | | | |  | | |  | | |  | |  |  |  |
| Sex | | 1.97 | | | 1.23 | | | |  | | |  | | |  | |  |  |  |
| Origin | | -0.13 | | | 0.52 | | | |  | | |  | | |  | |  |  |  |
| Release | | -0.03 | | | 0.37 | | | |  | | |  | | |  | |  |  |  |
| Treatment | | 0.01 | | | 0.36 | | | |  | | |  | | |  | |  |  |  |
| Origin:Release | | -0.07 | | | 0.50 | | | |  | | |  | | |  | |  |  |  |
|  | |  | | |  | | | |  | | |  | | |  | |  |  |  |
| **B. Infectivity untreated birds only** | | | | | | | | | | | | | | | | |  |  |  |
| *Component models* | | df | | | logLik | | | | AICc | | | Delta | | | Weight | |  |  |  |
| Sex | | 4 | | | -24.74 | | | | 58.65 | | | 0 | | | 0.41 | |  |  |  |
| (Null) | | 3 | | | -26.92 | | | | 60.52 | | | 1.87 | | | 0.16 | |  |  |  |
| Release + Sex | | 5 | | | -24.72 | | | | 61.26 | | | 2.62 | | | 0.11 | |  |  |  |
| Sex+ Origin | | 5 | | | -24.73 | | | | 61.28 | | | 2.63 | | | 0.11 | |  |  |  |
| Sex+ Release+ Origin+ O:R | | 7 | | | -21.92 | | | | 61.45 | | | 2.8 | | | 0.1 | |  |  |  |
| Origin | | 4 | | | -26.84 | | | | 62.85 | | | 4.2 | | | 0.05 | |  |  |  |
| Release | | 4 | | | -26.91 | | | | 62.99 | | | 4.34 | | | 0.05 | |  |  |  |
|  | |  | | |  | | | |  | | |  | | |  | |  |  |  |
| *Model averaged coefficients* | | Estimate | | | Std. | | | | RI | | | n models | | |  | |  |  |  |
| (Intercept) | | -0.20 | | | 0.35 | | | |  | | |  | | |  | |  |  |  |
| Sex | | 1.58 | | | 0.87 | | | | 0.74 | | | 4 | | |  | |  |  |  |
| Release site | | 0.09 | | | 0.75 | | | | 0.26 | | | 3 | | |  | |  |  |  |
| Origin | | -0.09 | | | 0.74 | | | | 0.26 | | | 3 | | |  | |  |  |  |
| Origin: Release | | -3.77 | | | 2.18 | | | | 0.1 | | | 1 | | |  | |  |  |  |
|  | |  | | |  | | | |  | | |  | | |  | |  |  |  |
| **C. Peak parasitaemia** | |  | | |  | | | |  | | |  | | |  | |  |  |  |
| *Component models* | | *df* | | | *logLik* | | | | *AICc* | | | *Delta* | | | *Weight* | |  |  |  |
| Release+Date | | 5 | | | -36.74 | | | | 85.28 | | | 0 | | | 0.43 | |  |  |  |
| Release+Sex+Date | | 6 | | | -35.89 | | | | 87.3 | | | 2.02 | | | 0.16 | |  |  |  |
| Release+Treat+tDate | | 6 | | | -36.05 | | | | 87.45 | | | 2.17 | | | 0.15 | |  |  |  |
| Release+Origin+Date | | 6 | | | -36.6 | | | | 88.68 | | | 3.4 | | | 0.08 | |  |  |  |
| Date | | 4 | | | -40.07 | | | | 89.28 | | | 3.99 | | | 0.06 | |  |  |  |
| Release+Sex+Treat+Date | | 7 | | | -35.35 | | | | 90.19 | | | 4.91 | | | 0.04 | |  |  |  |
| Release+Sex+Origin+Date | | 7 | | | -35.69 | | | | 90.94 | | | 5.66 | | | 0.03 | |  |  |  |
| Release+Origin+Treat+Date | | 7 | | | -35.86 | | | | 91.13 | | | 5.85 | | | 0.02 | |  |  |  |
| Treat+Date | | 5 | | | -39.38 | | | | 91.39 | | | 6.11 | | | 0.02 | |  |  |  |
| Sex+Date | | 5 | | | -39.34 | | | | 91.61 | | | 6.33 | | | 0.02 | |  |  |  |
|  | |  | | |  | | | |  | | |  | | |  | |  |  |  |
| *Model averaged coefficients* | | *Estimate* | | | *SE* | | | |  | | |  | | |  | |  |  |  |
| (Intercept) | | -2.63 | | | 0.22 | | | |  | | |  | | |  | |  |  |  |
| Release | | -1.04 | | | 0.53 | | | |  | | |  | | |  | |  |  |  |
| Date | | -0.41 | | | 0.46 | | | |  | | |  | | |  | |  |  |  |
| Sex | | -0.13 | | | 0.35 | | | |  | | |  | | |  | |  |  |  |
| Treatment | | -0.11 | | | 0.30 | | | |  | | |  | | |  | |  |  |  |
| Origin | | 0.02 | | | 0.17 | | | |  | | |  | | |  | |  |  |  |
| **D. Peak parasitaemia untreated birds only** | | | |  | |  | |  | | | | |  |  |  |  |  |  |  |
| *Component models* | | *df* | | *logLik* | | *AICc* | | *Delta* | | | | | *Weight* |  |  |  |  |  |  |
| Release+Sex | | 6 | | -22.23 | | 62.1 | | 0 | | | | | 0.59 |  |  |  |  |  |  |
| Release | | 5 | | -25.72 | | 65.11 | | 3.01 | | | | | 0.13 |  |  |  |  |  |  |
| (Null) | | 4 | | -28.26 | | 66.63 | | 4.53 | | | | | 0.06 |  |  |  |  |  |  |
| Release+Sex+Origin | | 7 | | -21.96 | | 66.76 | | 4.66 | | | | | 0.06 |  |  |  |  |  |  |
| Sex | | 5 | | -26.22 | | 66.98 | | 4.88 | | | | | 0.05 |  |  |  |  |  |  |
| Release+Sex+Date | | 7 | | -22.08 | | 67.05 | | 4.95 | | | | | 0.05 |  |  |  |  |  |  |
| Date | | 5 | | -27.04 | | 68.05 | | 5.95 | | | | | 0.03 |  |  |  |  |  |  |
| Release+Date | | 6 | | -25 | | 68.22 | | 6.12 | | | | | 0.03 |  |  |  |  |  |  |
|  | |  | |  | |  | |  | | | | |  |  |  |  |  |  |  |
| *Model averaged coefficients* | | *Estimate* | | *SE* | | RI | | nmodels | | | | |  |  |  |  |  |  |  |
| (Intercept) | | -2.694 | | 0.2038 | |  | |  | | | | |  |  |  |  |  |  |  |
| Release_site | | -1.2219 | | 0.4069 | | 0.86 | | 5 | | | | |  |  |  |  |  |  |  |
| Sex | | -1.4036 | | 0.5365 | | 0.75 | | 4 | | | | |  |  |  |  |  |  |  |
| Origin | | -0.2739 | | 0.4237 | | 0.11 | | 3 | | | | |  |  |  |  |  |  |  |
| Date | | -0.4045 | | 0.4915 | | 0.06 | | 1 | | | | |  |  |  |  |  |  |  |
|  | |  | | |  | | | |  | | |  | | |  | |  |  |  |
| **E. Final parasitaemia** | |  | | |  | | | |  | | |  | | |  | |  |  |  |
| *Component models* | | *df* | | | *logLik* | | | | *AICc* | | | *Delta* | | | *Weight* | |  |  |  |
| (Null) | | 3 | | | -31.81 | | | | 69.8 | | | 0 | | | 0.39 | |  |  |  |
| Release | | 4 | | | -31.06 | | | | 71.38 | | | 1.58 | | | 0.18 | |  |  |  |
| Treat | | 4 | | | -31.3 | | | | 72.14 | | | 2.33 | | | 0.12 | |  |  |  |
| Origin | | 4 | | | -31.65 | | | | 72.66 | | | 2.86 | | | 0.09 | |  |  |  |
| Sex | | 4 | | | -31.45 | | | | 72.78 | | | 2.98 | | | 0.09 | |  |  |  |
| Release + Treat | | 5 | | | -30.58 | | | | 74.09 | | | 4.29 | | | 0.05 | |  |  |  |
| Release + Origin | | 5 | | | -30.92 | | | | 74.75 | | | 4.95 | | | 0.03 | |  |  |  |
| Release + Sex | | 5 | | | -30.69 | | | | 74.75 | | | 4.95 | | | 0.03 | |  |  |  |
| Origin + Treat | | 5 | | | -31.05 | | | | 75.22 | | | 5.42 | | | 0.03 | |  |  |  |
|  | |  | | |  | | | |  | | |  | | |  | |  |  |  |
| *Model averaged coefficients* | | Estimate | | | SE | | | |  | | |  | | |  | |  |  |  |
| (Intercept) | | -1.91 | | | 1.53 | | | |  | | |  | | |  | |  |  |  |
| Release | | -0.15 | | | 0.33 | | | |  | | |  | | |  | |  |  |  |
| Treatment | | -0.07 | | | 0.26 | | | |  | | |  | | |  | |  |  |  |
| Origin | | 0.02 | | | 0.18 | | | |  | | |  | | |  | |  |  |  |
| Sex | | 0.01 | | | 0.20 | | | |  | | |  | | |  | |  |  |  |
| **F. Final parasitaemia, untreated birds**  *Component models* | *df* | | *logLik* | | | | *AICc* | | | | *Delta* | | | | | *Weight* | | | |
| (Null) | | 4 | | | -19.04 | | | | | 48.45 | | | 0 | | | | 0.63 | |  |
| Release | | 5 | | | -18.10 | | | | | 50.72 | | | 2.27 | | | | 0.2 |  |  |
| Sex | | 5 | | | -18.39 | | | | | 52.27 | | | 3.83 | | | | 0.09 |  |  |
| Origin | | 5 | | | -19.00 | | | | | 52.71 | | | 4.27 | | | | 0.07 |  |  |
|  | |  | | |  | | | | |  | | |  | | | |  |  |  |
|  | | *Estimate* | | | *SE* | | | | | *RI* | | | n models | | | |  |  |  |
| (Intercept) | | -2.77 | | | 0.21 | | | | |  | | |  | | | |  |  |  |
| Release | | -0.58 | | | 0.41 | | | | | 0.2 | | | 1 | | | |  |  |  |
| Sex | | -0.42 | | | 0.60 | | | | | 0.093 | | | 1 | | | |  |  |  |
| Origin | | 0.11 | | | 0.41 | | | | | 0.073 | | | 1 | | | |  |  |  |
