# Supplementary material for: Testing Local Adaptation in a Natural Great Tit-Malaria System: An Experimental Approach
Source: PLoS One. 2015 Nov 10;10(11):e0141391. doi: 10.1371/journal.pone.0141391 (PMC4640884; doi:10.1371/journal.pone.0141391)
Supplement: S3 File — (DOCX) [file pone.0141391.s003.docx]

| **File S3.** Models of host variables at the infection peak | | | | | |
| --- | --- | --- | --- | --- | --- |
| **A.Standardised mass index** |  |  |  |  |  |
| *Component models* | *df* | *logLik* | *AICc* | *Delta* | *Weight* |
| Date +Start SMI | 7 | -59.28 | 128.75 | 0 | 0.17 |
| Release +Date +Start SMI | 8 | -58.9 | 129.25 | 0.5 | 0.13 |
| Treat +Date +Start SMI | 8 | -59.33 | 130.12 | 1.37 | 0.08 |
| Sex +Date +Start SMI | 8 | -59.51 | 130.44 | 1.69 | 0.07 |
| Release +Treat +Date +Start SMI | 9 | -58.97 | 130.71 | 1.96 | 0.06 |
| Release +Sex +Date +Start SMI | 9 | -59.21 | 131.18 | 2.44 | 0.05 |
| Infection+Date +Start SMI | 8 | -59.91 | 131.2 | 2.45 | 0.05 |
| Origin +Date +Start SMI | 8 | -60.03 | 131.5 | 2.76 | 0.04 |
| Sex +Treat +Date +Start SMI | 9 | -59.47 | 131.77 | 3.03 | 0.04 |
| Infection+Release +Date +Start SMI | 9 | -59.57 | 131.92 | 3.17 | 0.03 |
| Release +Origin +Date +Start SMI | 9 | -59.67 | 132.07 | 3.32 | 0.03 |
| Infection+Sex +Date +Start SMI | 9 | -59.87 | 132.53 | 3.78 | 0.03 |
| Release +Sex +Treat +Date +Start SMI | 10 | -59.21 | 132.59 | 3.85 | 0.02 |
| Infection+Treat +Date +Start SMI | 9 | -59.97 | 132.7 | 3.95 | 0.02 |
| Origin +Treat +Date +Start SMI | 9 | -60.05 | 132.85 | 4.11 | 0.02 |
| Sex +Origin +Date +Start SMI | 9 | -60.26 | 133.32 | 4.57 | 0.02 |
| Infection+Release +Treat +Date +Start SMI | 10 | -59.66 | 133.52 | 4.77 | 0.02 |
| Infection+Release +Sex +Date +Start SMI | 10 | -59.66 | 133.57 | 4.83 | 0.01 |
| Release +Origin +Treat +Date +Start SMI | 10 | -59.71 | 133.6 | 4.85 | 0.01 |
| Infection+Release +Date +Start SMI+Inf:Rel | 10 | -59.14 | 133.73 | 4.98 | 0.01 |
| Infection+Sex +Treat +Date +Start SMI | 10 | -59.85 | 133.94 | 5.2 | 0.01 |
| Infection+Origin +Date +Start SMI | 9 | -60.65 | 134.08 | 5.33 | 0.01 |
| Release +Sex +Origin +Date +Start SMI | 10 | -59.97 | 134.15 | 5.4 | 0.01 |
| Sex +Origin +Treat +Date +Start SMI | 10 | -60.21 | 134.61 | 5.87 | 0.01 |
| Infection+Release +Origin +Date +Start SMI | 10 | -60.33 | 134.85 | 6.1 | 0.01 |
| Release +Origin +Date +Start SMI+Rel:Orig | 10 | -59.73 | 135.01 | 6.27 | 0.01 |
| Infection+Release +Sex +Treat +Date +Start SMI | 11 | -59.67 | 135.14 | 6.39 | 0.01 |
| Infection+Release +Treat +Date +Start SMI+Inf:Rel | 11 | -59.21 | 135.39 | 6.64 | 0.01 |
| Infection+Sex +Origin +Date +Start SMI | 10 | -60.62 | 135.53 | 6.79 | 0.01 |
|  |  |  |  |  |  |
| *Model averaged coefficients* | Estimate | SE |  |  |  |
| (Intercept) | 17.80 | 0.09 |  |  |  |
| Date | 0.27 | 0.20 |  |  |  |
| Start SMI | 0.89 | 0.20 |  |  |  |
| Release site | -0.29 | 0.20 |  |  |  |
| Treatment | -0.23 | 0.20 |  |  |  |
| Sex | 0.20 | 0.20 |  |  |  |
| Infection | -0.10 | 0.19 |  |  |  |
| Origin | 0.03 | 0.19 |  |  |  |
| Infection: Release | -0.38 | 0.36 |  |  |  |
| Origin:Release | 0.06 | 0.37 |  |  |  |
|  |  |  |  |  |  |
| **B. Temperature** |  |  |  |  |  |
| *Component models* | df | logLik | AICc | Delta | Weight |
| Infection + Treat | 6 | -57.25 | 122.5 | 0 | 0.15 |
| Treat | 5 | -57.7 | 122.7 | 0.21 | 0.13 |
| Infection + Release + Treat + Inf:Rel | 8 | -55.84 | 123.49 | 0.99 | 0.09 |
| Infection + Release + Origin + Treat + Inf:Rel + Inf:Orig + Rel: Orig+ Inf:Rel:Orig | 12 | -51.3 | 123.54 | 1.04 | 0.09 |
| Origin + Treat | 6 | -58 | 124.53 | 2.03 | 0.05 |
| Infection + Origin + Treat | 7 | -57.66 | 124.55 | 2.06 | 0.05 |
| Infection + Sex + Treat | 7 | -57.84 | 124.78 | 2.29 | 0.05 |
| Infection + Release + Treat | 7 | -57.68 | 125.07 | 2.57 | 0.04 |
| Infection + Release + Origin + Treat + Inf:Rel | 9 | -56.08 | 125.21 | 2.71 | 0.04 |
| Sex + Treat | 6 | -58.5 | 125.21 | 2.72 | 0.04 |
| Release + Treat | 6 | -58.15 | 125.22 | 2.72 | 0.04 |
| Infection + Release + Sex + Treat + Inf:Rel | 9 | -56.33 | 125.72 | 3.22 | 0.03 |
| Infection | 5 | -59.24 | 125.74 | 3.24 | 0.03 |
| (Null) | 4 | -59.96 | 126.39 | 3.9 | 0.02 |
| Infection + Release + Inf:Rel | 7 | -57.82 | 126.63 | 4.14 | 0.02 |
| Infection + Release + Sex + Origin + Treat + Inf:Rel + Inf:Orig + Rel: Orig+ Inf:Rel:Orig | 13 | -52.01 | 126.72 | 4.22 | 0.02 |
| Infection + Sex + Origin + Treat | 8 | -58.24 | 126.95 | 4.45 | 0.02 |
| Infection + Origin + Treat + Inf:Orig | 8 | -57.68 | 126.97 | 4.47 | 0.02 |
| Infection + Release + Origin + Treat + Inf:Rel + Inf:Orig | 10 | -55.76 | 127 | 4.5 | 0.02 |
| Release + Origin + Treat | 7 | -58.43 | 127.11 | 4.61 | 0.01 |
| Sex + Origin + Treat | 7 | -58.81 | 127.14 | 4.65 | 0.01 |
| Infection + Release + Origin + Treat | 8 | -58.08 | 127.19 | 4.69 | 0.01 |
| Infection + Release + Sex + Treat | 8 | -58.28 | 127.47 | 4.98 | 0.01 |
| Infection + Release + Sex + Origin + Treat + Inf:Rel | 10 | -56.56 | 127.57 | 5.08 | 0.01 |
|  |  |  |  |  |  |
| *Model averaged coefficients* | Estimate | SE |  |  |  |
| (Intercept) | -0.01 | 0.13 |  |  |  |
| Infection | -0.20 | 0.20 |  |  |  |
| Treatment | -0.43 | 0.20 |  |  |  |
| Release | 0.04 | 0.17 |  |  |  |
| Infection:Release | 0.23 | 0.40 |  |  |  |
| Origin | 0.06 | 0.14 |  |  |  |
| Inf:Origin | 0.04 | 0.16 |  |  |  |
| Origin:Release | -0.02 | 0.11 |  |  |  |
| Inf:Origin:Release | -0.20 | 0.61 |  |  |  |
| Sex | 0.02 | 0.09 |  |  |  |
|  |  |  |  |  |  |
| **C. Haematocrit** |  |  |  |  |  |
| *Models* | df | logLik | AICc | Delta | Weight |
| Sex+start Haem | 5 | 93.11 | 198.62 | 0 | 0.13 |
| Sex+Origin+start Haem | 6 | 90.49 | 198.42 | 0.19 | 0.12 |
| start Haem | 4 | 95.34 | 198.05 | 0.56 | 0.1 |
| Origin+start Haem | 5 | 92.72 | 197.88 | 0.74 | 0.09 |
| Release+Sex+start Haem | 6 | 89.72 | 196.78 | 1.83 | 0.05 |
| Sex+Treat+start Haem | 6 | 89.84 | 196.75 | 1.87 | 0.05 |
| Release+Sex+Origin+start Haem | 7 | 87.05 | 196.39 | 2.23 | 0.04 |
| Release+start Haem | 5 | 91.99 | 196.34 | 2.28 | 0.04 |
| Infection+Sex+start Haem | 6 | 89.48 | 196.15 | 2.47 | 0.04 |
| Sex+Origin+Treat+start Haem | 7 | 87.07 | 196.13 | 2.49 | 0.04 |
| Release+Origin+start Haem | 6 | 89.32 | 195.98 | 2.64 | 0.03 |
| Treat+start Haem | 5 | 91.93 | 195.97 | 2.64 | 0.03 |
| Infection+Sex+Origin+start Haem | 7 | 86.87 | 195.87 | 2.75 | 0.03 |
| Infection+start Haem | 5 | 91.68 | 195.69 | 2.92 | 0.03 |
| Origin+Treat+start Haem | 6 | 89.22 | 195.48 | 3.14 | 0.03 |
| Infection+Origin+start Haem | 6 | 89.05 | 195.4 | 3.22 | 0.03 |
| Release+Sex+Treat+start Haem | 7 | 86.45 | 194.81 | 3.8 | 0.02 |
| Release+Sex+Origin+start Haem+Rel: Orig | 8 | 84.36 | 194.35 | 4.27 | 0.02 |
| InfectionReleaseSex+start Haem | 7 | 86.11 | 194.22 | 4.4 | 0.01 |
| Release+Treat+start Haem | 6 | 88.59 | 194.16 | 4.45 | 0.01 |
| Infection+Sex+Treat+start Haem | 7 | 86.21 | 194.15 | 4.46 | 0.01 |
| Release+Sex+Origin+Treat+start Haem | 8 | 83.64 | 193.99 | 4.63 | 0.01 |
| Release+Origin+start Haem+Rel: Orig | 7 | 86.58 | 193.9 | 4.72 | 0.01 |
| Infection+Release+start Haem | 6 | 88.33 | 193.86 | 4.76 | 0.01 |
|  |  |  |  |  |  |
| *Model averaged coefficients* | Estimate | SE |  |  |  |
| (Intercept) | 0.47 | 0.00 |  |  |  |
| Sex | 0.02 | 0.01 |  |  |  |
| Haematocrit start | 0.03 | 0.01 |  |  |  |
| Origin | -0.01 | 0.01 |  |  |  |
| Release | 0.01 | 0.01 |  |  |  |
| Treatment | -0.01 | 0.01 |  |  |  |
| Infection | 0.00 | 0.01 |  |  |  |
| Origin: Release | -0.02 | 0.02 |  |  |  |
|  |  |  |  |  |  |
| **D. Oxidative stress** |  |  |  |  |  |
| *Component models* | df | logLik | AICc | Delta | Weight |
| (Null) | 4 | -144.54 | 300.26 | 0 | 0.16 |
| Infection | 5 | -142.5 | 301.05 | 0.79 | 0.11 |
| Release | 5 | -142.02 | 301.25 | 0.99 | 0.1 |
| Origin | 5 | -142.98 | 301.97 | 1.71 | 0.07 |
| Infection+Release | 6 | -140.01 | 302.17 | 1.91 | 0.06 |
| Sex | 5 | -143.14 | 302.53 | 2.27 | 0.05 |
| Treatment | 5 | -143.26 | 302.58 | 2.32 | 0.05 |
| Infection+Origin | 6 | -141.01 | 303 | 2.74 | 0.04 |
| Release+Origin | 6 | -140.46 | 303.07 | 2.81 | 0.04 |
| Infection+Origin+ Infection: Orig | 7 | -138.13 | 303.57 | 3.31 | 0.03 |
| Infection+Treatment | 6 | -141.27 | 303.6 | 3.33 | 0.03 |
| Infection + Sex | 6 | -141.17 | 303.64 | 3.37 | 0.03 |
| Release+ Sex | 6 | -140.63 | 303.65 | 3.39 | 0.03 |
| Release+Treatment | 6 | -140.76 | 303.7 | 3.43 | 0.03 |
| Release+Origin+Rel:Orig | 7 | -137.63 | 303.83 | 3.57 | 0.03 |
| Infection+Release+ Inf:Rel | 7 | -137.66 | 303.99 | 3.73 | 0.02 |
| Origin+Treatment | 6 | -141.55 | 304.17 | 3.91 | 0.02 |
| Infection+Release+Origin | 7 | -138.52 | 304.25 | 3.99 | 0.02 |
| Sex+Origin | 6 | -141.54 | 304.29 | 4.02 | 0.02 |
| Infection+Release+Treatment | 7 | -138.78 | 304.85 | 4.58 | 0.02 |
| Infection+Release+ Sex | 7 | -138.67 | 304.89 | 4.62 | 0.02 |
| Sex+Treatment | 6 | -141.86 | 305.03 | 4.76 | 0.01 |
| Infection+Release+Origin+ Inf: Orig | 8 | -135.72 | 305.13 | 4.87 | 0.01 |
|  |  |  |  |  |  |
| *Model averaged coefficients* | Estimate | SE |  |  |  |
| (Intercept) | 57.45 | 1.22 |  |  |  |
| Infection | -1.68 | 1.35 |  |  |  |
| Release | -2.88 | 2.42 |  |  |  |
| Origin | 1.14 | 1.31 |  |  |  |
| Sex | -0.56 | 1.50 |  |  |  |
| Treatment | -0.50 | 1.36 |  |  |  |
| Inf: Origin | 3.64 | 2.59 |  |  |  |
| Release: Origin | 3.62 | 2.59 |  |  |  |
| Inf: Release | 2.54 | 2.67 |  |  |  |
