# Supplementary material for: Testing Local Adaptation in a Natural Great Tit-Malaria System: An Experimental Approach
Source: PLoS One. 2015 Nov 10;10(11):e0141391. doi: 10.1371/journal.pone.0141391 (PMC4640884; doi:10.1371/journal.pone.0141391)
Supplement: S4 File — Table A SMI: standardized mass index, Table B: temperature. Table C: haematocrit. Table D: oxidative stress measured as membrane resistance. (DOCX) [file pone.0141391.s004.docx]

**File S4.** Models of host variables with parasitaemia at the infection peak

| **A. SMI** |  |  |  |  |  | |
| --- | --- | --- | --- | --- | --- | --- |
| *Component models* | *df* | *logLik* | *AICc* | *Delta* | *Weight* | |
| Release +Start SMI+Date | 7 | -26.21 | 67.4 | 0 | 0.25 | |
| Start SMI+Date | 6 | -27.46 | 67.63 | 0.23 | 0.22 | |
| Sex +Start SMI+Date | 7 | -26.57 | 68.8 | 1.4 | 0.12 | |
| Release +Sex +Start SMI+Date | 8 | -25.68 | 69.99 | 2.6 | 0.07 | |
| Release +Start SMI+Date +logr | 8 | -25.89 | 70.3 | 2.9 | 0.06 | |
| Origin +Start SMI+Date | 7 | -27.72 | 70.92 | 3.53 | 0.04 | |
| Treat +Start SMI+Date | 7 | -27.71 | 71.38 | 3.98 | 0.03 | |
| Start SMI+Date +logr | 7 | -27.75 | 71.42 | 4.03 | 0.03 | |
| Release +Origin +Start SMI+Date | 8 | -26.59 | 71.59 | 4.2 | 0.03 | |
| Release +Treat +Start SMI+Date | 8 | -26.49 | 71.63 | 4.24 | 0.03 | |
| Sex +Origin +Start SMI+Date | 8 | -26.9 | 72.78 | 5.38 | 0.02 | |
| Sex +Date | 6 | -29.36 | 72.83 | 5.43 | 0.02 | |
| Sex +Treat +Start SMI+Date | 8 | -26.79 | 72.88 | 5.48 | 0.02 | |
| Sex +Start SMI+Date +logr | 8 | -26.89 | 73.01 | 5.61 | 0.01 | |
| Date | 5 | -30.84 | 73.31 | 5.91 | 0.01 | |
| Release +Origin +Start SMI+Date +Orig:Rel | 9 | -24.9 | 73.32 | 5.92 | 0.01 | |
| Release +Sex +Start SMI+Date +logr | 9 | -25.57 | 73.95 | 6.56 | 0.01 | |
| Release +Sex +Treat +Start SMI+Date | 9 | -25.92 | 74.68 | 7.28 | 0.01 | |
| Release +Sex +Origin +Start SMI+Date | 9 | -26.07 | 74.77 | 7.37 | 0.01 | |
| Release +Treat +Start SMI+Date +logr | 9 | -26.11 | 74.95 | 7.55 | 0.01 | |
| Release +Origin +Start SMI+Date +logr | 9 | -26.25 | 74.98 | 7.58 | 0.01 | |
|  |  |  |  |  |  | |
| *Model averaged coefficients* | Estimate | Std. | RI | n models | |  |
| (Intercept) | 5.58 | 5.69 | - | - | |  |
| Release | -0.52 | 0.31 | 0.43 | 24 | |  |
| Start SMI | 0.81 | 0.27 | 1 | (fixed) | |  |
| Date | 0.42 | 0.29 | 1 | (fixed) | |  |
| Sex | 0.58 | 0.36 | 0.38 | 18 | |  |
| logr | -0.23 | 0.35 | 0.13 | 11 | |  |
| Origin | 0.10 | 0.28 | 0.11 | 13 | |  |
| Treatment | 0.01 | 0.31 | 0.08 | 6 | |  |
| Origin: Release | -1.01 | 0.64 | 0.01 | 1 |  | |

|  |  |  |  |  |  | |
| --- | --- | --- | --- | --- | --- | --- |
| **B. Temperature** |  |  |  |  |  | |
| *Component models* | *df* | *logLik* | *AICc* | *Delta* | *Weight* | |
| Date | 5 | -30.6 | 72.28 | 0 | 0.34 | |
| Release+Date | 6 | -30 | 73.85 | 1.58 | 0.15 | |
| Date+logr | 6 | -30.31 | 74.41 | 2.13 | 0.12 | |
| Origin+Date | 6 | -30.62 | 75.45 | 3.17 | 0.07 | |
| Treatment+Date | 6 | -30.73 | 75.5 | 3.22 | 0.07 | |
| Sex+Date | 6 | -30.64 | 75.68 | 3.41 | 0.06 | |
| Release+Origin+Date | 7 | -29.83 | 76.87 | 4.6 | 0.03 | |
| Release+Date+logr | 7 | -29.89 | 77.04 | 4.76 | 0.03 | |
| Release+Treatment+Date | 7 | -30.17 | 77.62 | 5.34 | 0.02 | |
| Release+Sex+Date | 7 | -30.03 | 77.64 | 5.37 | 0.02 | |
| Origin+Date+logr | 7 | -30.21 | 77.71 | 5.43 | 0.02 | |
| Treatment+Date+logr | 7 | -30.39 | 78.06 | 5.79 | 0.02 | |
| Sex+Date+logr | 7 | -30.3 | 78.16 | 5.88 | 0.02 | |
| Release+Origin+Date+Origin: Release | 8 | -28.37 | 78.68 | 6.41 | 0.01 | |
| Origin+Treatment+Date | 7 | -30.71 | 78.99 | 6.72 | 0.01 | |
|  |  |  |  |  |  | |
| *Model averaged coefficients* | Estimate | Std. | RI | n models | |  |
| (Intercept) | 41.43 | 0.19 | - | - | |  |
| July_daymax | -1.20 | 0.35 | 1 | (fixed) | |  |
| Release_site | 0.46 | 0.35 | 0.28 | 6 | |  |
| logr_max | -0.40 | 0.34 | 0.21 | 5 | |  |
| Site_origin | 0.21 | 0.35 | 0.15 | 5 | |  |
| Treat2 | -0.13 | 0.33 | 0.12 | 4 | |  |
| sex | -0.01 | 0.39 | 0.1 | 3 | |  |
| Release_site:Site_origin | -0.91 | 0.63 | 0.01 | 1 | |  |
| **C. Haematocrit** |  |  |  |  | |  |
| *Component models* | *df* | *logLik* | *AICc* | *Delta* | *Weight* | |
| Date | 6 | 44.02 | -85.79 | 0 | 0.38 | |
| Treatment+Date | 7 | 41.89 | -84.98 | 0.81 | 0.25 | |
| Sex+Date | 7 | 40.84 | -82.26 | 3.54 | 0.06 | |
| Origin+Date | 7 | 40.89 | -82.05 | 3.75 | 0.06 | |
| Date+logr | 7 | 40.66 | -81.99 | 3.8 | 0.06 | |
| Release+Date | 7 | 40.85 | -81.88 | 3.91 | 0.05 | |
| Treatment+Date+logr | 8 | 38.71 | -81.11 | 4.68 | 0.04 | |
| Origin+Treatment+Date | 8 | 38.81 | -81.01 | 4.78 | 0.03 | |
| Sex+Treatment+Date | 8 | 38.66 | -80.89 | 4.91 | 0.03 | |
| Release+Treatment+Date | 8 | 38.68 | -80.6 | 5.19 | 0.03 | |
| Sex+Origin+Date | 8 | 37.77 | -78.15 | 7.64 | 0.01 | |

|  |  |  |  |  |  | | |
| --- | --- | --- | --- | --- | --- | --- | --- |
| *Model averaged coefficients* | Estimate | Std. | RI | n models | |  | |
| (Intercept) | 0.48 | 0.01 | - | - | |  | |
| Date | 0.01 | 0.01 | 1 | (fixed) | |  | |
| Treat2 | 0.02 | 0.01 | 0.38 | 5 | |  | |
| Sex | 0.01 | 0.01 | 0.11 | 3 | |  | |
| Origin | -0.01 | 0.02 | 0.1 | 3 | |  | |
| logr | 0.01 | 0.01 | 0.09 | 2 | |  | |
| Release_site | 0.00 | 0.02 | 0.08 | 2 | |  | |
|  |  |  |  |  | | |  |
| **D. Oxidative stress** |  |  |  |  | | |  |
| *Component models* | *df* | *logLik* | *AICc* | *Delta* | | | *Weight* |
| Date | 5 | -64.34 | 149.13 | 0 | | | 0.36 |
| Origin + Date | 6 | -61.57 | 150.97 | 1.84 | | | 0.14 |
| Date+ logr | 6 | -61.67 | 151.41 | 2.28 | | | 0.12 |
| Sex +Date | 6 | -61.69 | 151.78 | 2.65 | | | 0.1 |
| Release +Date | 6 | -61.88 | 152.41 | 3.28 | | | 0.07 |
| Treat +Date | 6 | -62.38 | 153.1 | 3.97 | | | 0.05 |
| Sex +Origin + Date | 7 | -58.69 | 153.42 | 4.3 | | | 0.04 |
| Origin + Date+ logr | 7 | -58.93 | 153.72 | 4.59 | | | 0.04 |
| Sex +Date+ logr | 7 | -59.05 | 154.52 | 5.4 | | | 0.02 |
| Origin + Treat +Date | 7 | -59.36 | 155.06 | 5.93 | | | 0.02 |
| Release +Origin + Date | 7 | -59.25 | 155.17 | 6.04 | | | 0.02 |
| Release +Sex +Date | 7 | -59.06 | 155.35 | 6.22 | | | 0.02 |
| Release +Date+ logr | 7 | -59.38 | 155.84 | 6.71 | | | 0.01 |
|  |  |  |  |  | | |  |
| *Model averaged coefficients* | Estimate | SE | RI | n models | | |  |
| (Intercept) | 56.41 | 1.67 | - | - | | |  |
| Date | 1.11 | 2.56 | 1 | (fixed) | | |  |
| Origin | 0.89 | 1.96 | 0.26 | 5 | | |  |
| logr | 0.62 | 1.73 | 0.19 |  | | |  |
| Sex | -0.69 | 1.98 | 0.18 | 4 | | |  |
| Release | -0.30 | 1.45 | 0.12 | 4 | | |  |
| Treatment | -0.07 | 0.81 | 0.07 | 2 | | |  |
