# Supplementary material for: Testing Local Adaptation in a Natural Great Tit-Malaria System: An Experimental Approach
Source: PLoS One. 2015 Nov 10;10(11):e0141391. doi: 10.1371/journal.pone.0141391 (PMC4640884; doi:10.1371/journal.pone.0141391)
Supplement: S5 File — Table A SMI: standardized mass index, Table B: temperature. Table C: haematocrit. Table D: oxidative stress measured as membrane resistance. (DOCX) [file pone.0141391.s005.docx]

**File S5.** Models of host variables at the end of the experiment

| **A. SMI** |  |  |  |  |  |
| --- | --- | --- | --- | --- | --- |
| *Component models* | df | logLik | AICc | Delta | Weight |
| Infection+ Release+Infection: Rel+Inf:Rel | 7 | -54.25 | 116.47 | 0 | 0.39 |
| Release+Infection: Rel | 5 | -56.62 | 118.36 | 1.89 | 0.15 |
| Infection+ Release+Infection: Rel+Origin+Inf:Rel | 8 | -54.86 | 119.17 | 2.69 | 0.1 |
| Infection+ Release+Infection: Rel+Treat+Inf:Rel | 8 | -55.1 | 119.2 | 2.72 | 0.1 |
| Release+Infection: Rel+Origin | 6 | -57.09 | 120.62 | 4.15 | 0.05 |
| Release+Infection: Rel+Treat | 6 | -57.41 | 120.8 | 4.33 | 0.04 |
| Infection+ Release+Infection: Rel | 6 | -57.36 | 120.83 | 4.36 | 0.04 |
| Infection+ Release+Infection: Rel+Origin+Inf:Rel+Rel:Orig | 9 | -54.91 | 121.89 | 5.42 | 0.03 |
| Infection+ Release+Infection: Rel+Origin+Inf:Rel+Inf:Orig | 9 | -54.93 | 121.92 | 5.45 | 0.03 |
| Infection+ Release+Infection: Rel+Origin+Treat+Inf:Rel | 9 | -55.7 | 122 | 5.52 | 0.02 |
| Release+Infection: Rel+Origin+Treat | 7 | -57.87 | 123.14 | 6.67 | 0.01 |
| Infection+ Release+Infection: Rel+Origin | 7 | -57.82 | 123.22 | 6.75 | 0.01 |
| Release+Infection: Rel+Origin+Rel:Orig | 7 | -57.17 | 123.23 | 6.76 | 0.01 |
|  |  |  |  |  |  |
| *Model averaged coefficients* | Estimate | SE |  |  |  |
| (Intercept) | 17.81 | 0.11 |  |  |  |
| Infection | -0.02 | 0.18 |  |  |  |
| Release | 0.80 | 0.17 |  |  |  |
| Sex | 0.63 | 0.18 |  |  |  |
| Inf:Release | -0.91 | 0.35 |  |  |  |
| Origin | 0.07 | 0.22 |  |  |  |
| Treat | -0.02 | 0.17 |  |  |  |
| Origin:Release | -0.09 | 0.36 |  |  |  |
| Inf:Orig | -0.10 | 0.36 |  |  |  |
|  |  |  |  |  |  |
| **B. Temperature** |  |  |  |  |  |
| *Component models* | df | logLik | AICc | Delta | Weight |
| (Null) | 3 | -54.55 | 113.64 | 0 | 0.16 |
| Infection | 4 | -54.41 | 113.72 | 0.08 | 0.15 |
| Release | 4 | -54.49 | 115.36 | 1.71 | 0.07 |
| Infection+Release | 5 | -54.41 | 115.58 | 1.94 | 0.06 |
| Sex | 4 | -55.27 | 115.63 | 1.99 | 0.06 |
| Origin | 4 | -55.51 | 115.87 | 2.23 | 0.05 |
| Treat | 4 | -55.47 | 115.9 | 2.26 | 0.05 |
| Infection+Treat | 5 | -55.31 | 116.05 | 2.41 | 0.05 |
| Infection+Origin | 5 | -55.4 | 116.12 | 2.47 | 0.05 |
| Infection+Sex | 5 | -55.25 | 116.12 | 2.48 | 0.05 |
| Infection+Release+Inf:Rel | 6 | -54.04 | 116.78 | 3.14 | 0.03 |
| Release+Sex | 5 | -55.22 | 117.44 | 3.8 | 0.02 |
| Release+Origin | 5 | -55.46 | 117.68 | 4.04 | 0.02 |
| Release+Treat | 5 | -55.41 | 117.69 | 4.05 | 0.02 |
| Sex+Origin | 5 | -56.22 | 117.93 | 4.29 | 0.02 |
| Infection+Release+Treat | 6 | -55.31 | 117.98 | 4.34 | 0.02 |
| Sex+Treat | 5 | -56.18 | 117.98 | 4.34 | 0.02 |
| Infection+Release+Origin | 6 | -55.4 | 118.06 | 4.42 | 0.02 |
| Infection+Release+Sex | 6 | -55.24 | 118.06 | 4.42 | 0.02 |
| Origin+Treat | 5 | -56.41 | 118.25 | 4.61 | 0.02 |
| Infection+Sex+Treat | 6 | -56.14 | 118.54 | 4.9 | 0.01 |
| Infection+Origin+Treat | 6 | -56.28 | 118.55 | 4.91 | 0.01 |
| Infection+Origin+Inf:Orig | 6 | -55.55 | 118.56 | 4.91 | 0.01 |
| Infection+Sex+Origin | 6 | -56.22 | 118.61 | 4.97 | 0.01 |
|  |  |  |  |  |  |
| *Model averaged coefficients* | Estimate | SE |  |  |  |
| (Intercept) | 41.67 | 0.15 |  |  |  |
| Infection | -0.23 | 0.16 |  |  |  |
| Release | -0.24 | 0.31 |  |  |  |
| Sex | -0.06 | 0.17 |  |  |  |
| Origin | 0.03 | 0.15 |  |  |  |
| Treatment | 0.04 | 0.16 |  |  |  |
| Infection:Release | 0.35 | 0.31 |  |  |  |
| Inf:Origin | 0.09 | 0.33 |  |  |  |
|  |  |  |  |  |  |
| **C. Haematocrit** |  |  |  |  |  |
| *Component models* | df | logLik | AICc | Delta | Weight |
| Infection+ReleaseHaem start +Inf:Rel | 8 | 71.65 | 160.59 | 0 | 0.13 |
| Infection + Release + Haem start | 7 | 72.9 | 159.97 | 0.62 | 0.09 |
| Release + Haem start | 6 | 75.3 | 159.82 | 0.77 | 0.09 |
| Infection+Release+Origin+Haem start +Inf:Rel+Inf:Orig +Rel:Orig+Inf:Rel:Orig | 12 | 64.6 | 159.78 | 0.81 | 0.08 |
| Infection+Release+Origin+Haem start +Inf:Rel+Inf:Orig +Rel:Orig | 11 | 64.91 | 159.63 | 0.96 | 0.08 |
| Infection+Release+Origin+Treat +Haem start +Inf:Rel+Inf:Orig | 11 | 64.05 | 158.93 | 1.66 | 0.05 |
| Infection+Release+Treat +Haem start +Inf:Rel | 9 | 68.39 | -158.6 | 1.99 | 0.05 |
| Release+Origin+Haem start | 7 | 72.42 | 158.42 | 2.17 | 0.04 |
| Infection+Release+Origin+Haem start +Inf:Rel | 9 | 68.62 | 158.39 | 2.2 | 0.04 |
| Infection+Release+Treat +Haem start | 8 | 69.9 | 158.29 | 2.3 | 0.04 |
| Infection+Release+Origin+Haem start +Inf:Rel+Inf:Orig | 10 | 66.53 | 158.09 | 2.5 | 0.04 |
| Infection+ReleaseOrigin+Haem start | 8 | 69.83 | -157.8 | 2.79 | 0.03 |
| Release+Sex+Haem start | 7 | 71.88 | 157.76 | 2.83 | 0.03 |
| Infection+ReleaseSex+Haem start | 8 | 69.38 | 157.73 | 2.86 | 0.03 |
| Infection+ReleaseSex+Haem start +Inf:Rel | 9 | 67.89 | 157.67 | 2.92 | 0.03 |
| Release+Treat+Haem start | 7 | 72.08 | 157.65 | 2.95 | 0.03 |
| Infection+ReleaseOrigin+Treat +Haem start +Inf:Orig | 10 | 65.35 | 157.35 | 3.24 | 0.02 |
| Infection+ReleaseOrigin+Treat +Haem start +Inf:Rel+Inf:Orig +Rel:Orig | 12 | 61.48 | 156.72 | 3.87 | 0.02 |
| Infection+ReleaseSex+Treat +Haem start | 9 | 66.53 | 156.45 | 4.14 | 0.02 |
| Infection+ReleaseOrigin+Haem start +Inf:Orig | 9 | 67.51 | 156.38 | 4.21 | 0.02 |
| ReleaseSex+Origin+Haem start | 8 | 69.03 | 156.24 | 4.35 | 0.01 |
| Infection+ReleaseOrigin+Haem start +Inf:Rel+Rel:Orig | 10 | 66.07 | 156.18 | 4.41 | 0.01 |
| ReleaseSex+Treat +Haem start | 8 | 68.92 | 156.09 | 4.5 | 0.01 |
| Infection+Release+Origin+Treat +Haem start +Inf:Rel | 10 | 65.24 | 155.95 | 4.65 | 0.01 |
|  |  |  |  |  |  |
| *Model averaged coefficients* | Estimate | SE |  |  |  |
| (Intercept) | 0.48 | 0.01 |  |  |  |
| Infection | -0.02 | 0.01 |  |  |  |
| Release site | -0.03 | 0.01 |  |  |  |
| Haematocrit start | 0.03 | 0.01 |  |  |  |
| Infection : Release site | 0.04 | 0.02 |  |  |  |
| Origin | 0.01 | 0.01 |  |  |  |
| Infection: Origin | -0.03 | 0.01 |  |  |  |
| Release: Origin | 0.03 | 0.02 |  |  |  |
| Infection: Release: Origin | -0.08 | 0.04 |  |  |  |
| Treatment | 0.01 | 0.01 |  |  |  |
| Sex | -0.01 | 0.01 |  |  |  |
|  |  |  |  |  |  |
| **D. Oxidative stress** |  |  |  |  |  |
| *Component models* | df | logLik | AICc | Delta | Weight |
| Sex+Origin | 6 | 132.68 | 284.08 | 0 | 0.13 |
| Origin | 5 | 134.96 | 284.36 | 0.28 | 0.11 |
| Sex | 5 | 135.05 | 284.77 | 0.69 | 0.09 |
| (Null) | 4 | 137.55 | 285.49 | 1.4 | 0.06 |
| Release+Sex+Origin | 7 | 130.7 | 285.6 | 1.51 | 0.06 |
| Release+Origin | 6 | 133.04 | 285.91 | 1.82 | 0.05 |
| Sex+Origin+Treat | 7 | 131.51 | 286.04 | 1.95 | 0.05 |
| Release+Sex | 6 | 133.06 | 286.21 | 2.12 | 0.04 |
| Origin+Treat | 6 | 133.84 | 286.37 | 2.28 | 0.04 |
| Infection+Origin | 6 | 133.92 | 286.7 | 2.62 | 0.03 |
| Infection+Sex+Origin | 7 | 131.74 | 286.77 | 2.68 | 0.03 |
| Release | 5 | -135.62 | 286.94 | 2.86 | 0.03 |
| Sex+Treat | 6 | -134.14 | 287.14 | 3.06 | 0.03 |
| Infection+Sex | 6 | -134.1 | 287.32 | 3.24 | 0.03 |
| Release+Sex+Origin+Treat | 8 | -129.54 | 287.66 | 3.58 | 0.02 |
| Treat | 5 | -136.65 | 287.83 | 3.75 | 0.02 |
| Infection | 5 | -136.57 | 287.88 | 3.8 | 0.02 |
| Release+Origin+Treat | 7 | -131.93 | 288.09 | 4.01 | 0.02 |
| Infection+Release+Origin | 7 | -131.99 | 288.33 | 4.25 | 0.02 |
| Infection+Release+Sex+Origin | 8 | -129.75 | 288.39 | 4.31 | 0.02 |
| Release+Sex+Origin+Rel:Orig | 8 | -129.17 | 288.4 | 4.31 | 0.01 |
| Infection+Origin+Inf:Orig | 7 | -131.9 | 288.48 | 4.4 | 0.01 |
| Release+Origin+Rel:Orig | 7 | -131.49 | 288.57 | 4.49 | 0.01 |
| Release+Sex+Treat | 7 | -132.15 | 288.67 | 4.59 | 0.01 |
| Infection+Origin+Treat | 7 | -132.76 | 288.76 | 4.67 | 0.01 |
| Infection+Sex+Origin+Treat | 8 | -130.56 | 288.83 | 4.75 | 0.01 |
| Infection+Release+Sex | 7 | -132.11 | 288.86 | 4.78 | 0.01 |
|  |  |  |  |  |  |
| *Model averaged coefficients* | Estimate | SE |  |  |  |
| (Intercept) | 57.67 | 0.85 |  |  |  |
| Sex | 1.73 | 1.00 |  |  |  |
| Origin | 1.71 | 0.92 |  |  |  |
| Release | -1.72 | 1.69 |  |  |  |
| Treatment | -0.62 | 0.94 |  |  |  |
| Infection | 0.24 | 1.05 |  |  |  |
| Origin:Release | -0.03 | 1.86 |  |  |  |
| Inf:Origin | 1.82 | 1.97 |  |  |  |
