# Supplementary material for: Testing Local Adaptation in a Natural Great Tit-Malaria System: An Experimental Approach
Source: PLoS One. 2015 Nov 10;10(11):e0141391. doi: 10.1371/journal.pone.0141391 (PMC4640884; doi:10.1371/journal.pone.0141391)
Supplement: S6 File — Table A SMI: standardized mass index, Table B: temperature. Table C: haematocrit. Table D: oxidative stress measured as membrane resistance. (DOCX) [file pone.0141391.s006.docx]

**File S6** Models of host variables, explicitly accounting for parasitaemia at the end of the experiment

| **A. SMI** |  |  |  |  |  | | |
| --- | --- | --- | --- | --- | --- | --- | --- |
| *Component models* | df | logLik | AICc | Delta | Weight | | |
| Start SMI | 5 | -17.91 | 45.96 | 0 | 0.31 | | |
| Sex+ Start SMI | 6 | -16.82 | 46.67 | 0.71 | 0.21 | | |
| Release+Origin+ Start SMI+Origin:Release | 8 | -13.88 | 47.88 | 1.92 | 0.12 | | |
| Release+ Start SMI | 6 | -17.78 | 48.57 | 2.61 | 0.08 | | |
| Treatment+ Start SMI | 6 | -18.18 | 49.34 | 3.38 | 0.06 | | |
| Origin+ Start SMI | 6 | -18.28 | 49.82 | 3.86 | 0.04 | | |
| logr+ Start SMI | 6 | -18.5 | 49.82 | 3.86 | 0.04 | | |
| Release+Sex+ Start SMI | 7 | -17.09 | 50.8 | 4.84 | 0.03 | | |
| Sex+logr+ Start SMI | 7 | -17.42 | 50.88 | 4.92 | 0.03 | | |
| Sex+Origin+ Start SMI | 7 | -17.17 | 50.92 | 4.96 | 0.03 | | |
| Sex+Treatment+ Start SMI | 7 | -17.3 | 51.05 | 5.09 | 0.02 | | |
|  |  |  |  |  |  | | |
| *Model averaged coefficients* | Estimate | SE | RI | n models | | |  |
| (Intercept) | 17.8353 | 0.13439 | - | - | | |  |
| Start SMI | 0.87323 | 0.28701 | 1 | (fixed) | | |  |
| Sex | 0.4882 | 0.29434 | 0.32 | 5 | | |  |
| Release site | -0.2383 | 0.25224 | 0.26 | 6 | | |  |
| Site origin | -0.0943 | 0.24782 | 0.21 | 5 | | |  |
| Release site:Site origin | -1.443 | 0.44546 | 0.13 | 2 | | |  |
| Treatment | 0.13558 | 0.2486 | 0.09 | 3 | | |  |
| logr | 0.06235 | 0.22102 | 0.08 | 3 | | |  |
|  |  |  |  |  |  | | |
| **B. Temperature** |  |  |  |  |  | | |
| *Component models* | df | logLik | AICc | Delta | Weight | | |
| logr | 4 | -24.24 | 56.65 | 0 | 0.27 | | |
| Treatment+logr | 5 | -23.32 | 57.46 | 0.81 | 0.18 | | |
| Treatment | 4 | -25.13 | 58.77 | 2.13 | 0.09 | | |
| (Null) | 3 | -26.5 | 59.05 | 2.4 | 0.08 | | |
| Sex+logr | 5 | -24.21 | 59.84 | 3.19 | 0.05 | | |
| Release+logr | 5 | -24.22 | 59.91 | 3.26 | 0.05 | | |
| Origin+logr | 5 | -24.53 | 60.03 | 3.39 | 0.05 | | |
| Sex+Treatment+logr | 6 | -23.24 | 60.88 | 4.23 | 0.03 | | |
| Release+Treatment+logr | 6 | -23.23 | 60.99 | 4.35 | 0.03 | | |
| Origin+Treatment+logr | 6 | -23.57 | 61.06 | 4.41 | 0.03 | | |
| Sex+Treatment | 5 | -25 | 61.74 | 5.09 | 0.02 | | |
| Sex+ | 4 | -26.38 | 61.82 | 5.17 | 0.02 | | |
| Origin+Treatment | 5 | -25.31 | 61.93 | 5.28 | 0.02 | | |
| Origin+ | 4 | -26.73 | 62.05 | 5.4 | 0.02 | | |
| Release+ | 4 | -26.42 | 62.11 | 5.47 | 0.02 | | |
| Release+Treatment | 5 | -25.03 | 62.15 | 5.51 | 0.02 | | |
| Release+Sex+logr | 6 | -24.15 | 63.49 | 6.85 | 0.01 | | |
|  |  |  |  |  |  | | |
| *Model averaged coefficients* | Estimate | SE | RI | n models | | |  |
| (Intercept) | 41.41 | 0.19 | - | - | | |  |
| logr | -0.69 | 0.31 | 0.71 | 9 | | |  |
| Treat2 | 0.53 | 0.32 | 0.43 | 8 | | |  |
| sex | -0.19 | 0.38 | 0.14 | 5 | | |  |
| Release_site | -0.13 | 0.41 | 0.13 | 5 | | |  |
| Site origin | -0.07 | 0.31 | 0.12 | 4 | | |  |
|  |  |  |  |  |  | | |
| **C. Haematocrit** |  |  |  |  |  | | |
| *Component models* | df | logLik | AICc | Delta | Weight | | |
| (Null) | 3 | 29.22 | -57.86 | 0 | 0.32 | | |
| Release | 4 | 27.11 | -56.04 | 1.82 | 0.13 | | |
| Treatment | 4 | 26.68 | -55.95 | 1.91 | 0.12 | | |
| Release+Treatment | 5 | 25.24 | -55.49 | 2.38 | 0.1 | | |
| Sex | 4 | 25.92 | -54.55 | 3.31 | 0.06 | | |
| Origin | 4 | 26.33 | -54.4 | 3.46 | 0.06 | | |
| logr | 4 | 25.99 | -54.25 | 3.62 | 0.05 | | |
| Sex+Treatment | 5 | 24.25 | -53.72 | 4.14 | 0.04 | | |
| Release+Sex+Treatment | 6 | 22.84 | -52.35 | 5.51 | 0.02 | | |
| Release+logr | 5 | 24.14 | -52.22 | 5.64 | 0.02 | | |
| Release+Sex | 5 | 23.78 | -52.04 | 5.82 | 0.02 | | |
| Treatment+logr | 5 | 23.66 | -51.85 | 6.01 | 0.02 | | |
| Release+Origin | 5 | 24.17 | -51.78 | 6.08 | 0.02 | | |
| Origin+Treatment | 5 | 23.66 | -51.6 | 6.26 | 0.01 | | |
| Release+Origin+Origin:Release | 6 | 23.54 | -50.84 | 7.02 | 0.01 | | |
|  |  |  |  |  |  | | |
| *Model averaged coefficients* | Estimate | SE | RI | n models | |  | |
| (Intercept) | 0.48 | 0.01 | - | - | |  | |
| Release_site | -0.03 | 0.02 | 0.314 | 6 | |  | |
| Treat2 | 0.03 | 0.02 | 0.311 | 7 | |  | |
| sex | -0.01 | 0.02 | 0.141 | 4 | |  | |
| Site_origin | 0.01 | 0.02 | 0.096 | 4 | |  | |
| logr | 0.00 | 0.02 | 0.088 | 3 | |  | |
| Release_site:Site_origin | -0.08 | 0.04 | <0.01 | 1 | |  | |
|  |  |  |  |  |  | | |
| **D. Oxidative stress** |  |  |  |  |  | | |
| *Component models* | df | logLik | AICc | Delta | Weight | | |
| Sex | 4 | -51.33 | 118.22 | 0 | 0.18 | | |
| (Null) | 3 | -54.39 | 118.53 | 0.3 | 0.16 | | |
| Origin | 4 | -52.05 | 119.49 | 1.26 | 0.1 | | |
| Sex+Origin | 5 | -49.38 | 120.15 | 1.92 | 0.07 | | |
| Release | 4 | -51.94 | 120.27 | 2.05 | 0.07 | | |
| Treat | 4 | -52.47 | 120.47 | 2.24 | 0.06 | | |
| Origin+Treat | 5 | -49.58 | 120.61 | 2.38 | 0.06 | | |
| Release+Sex | 5 | -49 | 120.78 | 2.56 | 0.05 | | |
| Sex+Treat | 5 | -49.66 | 120.93 | 2.71 | 0.05 | | |
| logr | 4 | -52.91 | 121.45 | 3.23 | 0.04 | | |
| Sex+logr | 5 | -50.07 | 121.84 | 3.61 | 0.03 | | |
| Release+Origin | 5 | -49.73 | 121.84 | 3.62 | 0.03 | | |
| Sex+Origin+Treat | 6 | -47.14 | 122.25 | 4.03 | 0.02 | | |
| Origin+logr | 5 | -50.42 | 122.56 | 4.33 | 0.02 | | |
| Release+Treat | 5 | -50.1 | 122.73 | 4.51 | 0.02 | | |
| Release+Sex+Origin | 6 | -47.13 | 123.31 | 5.09 | 0.01 | | |
| Release+logr | 5 | -50.48 | 123.65 | 5.42 | 0.01 | | |
| Release+Origin+Treat | 6 | -47.42 | 123.75 | 5.52 | 0.01 | | |
| Treat+logr | 5 | -50.97 | 123.97 | 5.75 | 0.01 | | |
| Release+Sex+Treat | 6 | -47.4 | 124.03 | 5.81 | 0.01 | | |
|  |  |  |  |  |  | | |
| *Model averaged coefficients* | Estimate | Std. | RI | n models | |  | |
| (Intercept) | 48.86 | 21.06 |  |  | |  | |
| Sex | 1.26 | 1.78 | 0.43 | 8 | |  | |
| Origin | 0.69 | 1.32 | 0.32 | 8 | |  | |
| Release | -0.54 | 1.54 | 0.21 | 8 | |  | |
| Treatment | -0.44 | 1.10 | 0.24 | 8 | |  | |
| logr | 0.08 | 0.58 | 0.11 | 5 | |  | |
